# Understanding Ion Intercalation Characteristics of Layered Materials in Superconcentrated Electrolytes: Effects of Concentration, Temperature, and Anion Identity

**DOI:** 10.1021/acsomega.5c06741

**Published:** 2025-09-04

**Authors:** Sirintra Arayawate, Napaporn Kareeklin, Thanit Saisopa, Prayoon Songsiriritthigul, Pawin Iamprasertkun

**Affiliations:** † School of Bio-Chemical Engineering and Technology, Sirindhorn International Institute of Technology, 37698 Thammasat University, Khlong Nueng 12120, Thailand; ‡ Research Unit in Sustainable Electrochemical Intelligent, Thammasat University, Khlong Nueng 12120, Thailand; § Department of Applied Physics, Faculty of Sciences and Liberal Arts, 202946 Rajamangala University of Technology Isan, Nakhon Ratchasima, 30000, Thailand; ∥ School of Physics, 65162 Suranaree University of Technology, Nakhon Ratchasima, 30000, Thailand

## Abstract

Transition metal dichalcogenides (TMDs) are promising layered materials for energy storage applications, due to their ability to host ions within their interlayer galleries. Reducing the hydration shell of aqueous ions, which can be achieved through high-concentration electrolytes, enhances ion intercalation, with some systems reaching the “water-in-salt” regime. Understanding the interplay among ion identity, concentration, and interlayer electrochemistry is thus essential for advancing high-performance energy storage devices. In this study, exfoliated molybdenum disulfide (MoS_2_) was employed as a model TMD electrode to investigate these effects. A systematic evaluation was conducted using lithium-based electrolytes (LiTFSI, LiNO_3_, Li_2_SO_4_, and LiCl) across a wide concentration range, from conventional “salt-in-water” to superconcentrated “water-in-salt” conditions. Electrochemical performance was further analyzed under various temperatures (−5 to 60 °C). The 5 m LiTFSI system demonstrated the highest specific capacitance (205 F g^–1^ at 5 mV s^–1^) and optimal ion mobility under ambient conditions. Temperature-dependent behavior revealed distinct operational windows for each concentration, with the 5 m electrolyte excelling at low-to-moderate temperatures and the 20 m system becoming favorable at elevated temperatures due to enhanced ion dynamics. Among the salts, LiTFSI provided the widest electrochemical stability window and fastest relaxation behavior, while LiNO_3_ emerged as a cost-effective alternative with comparable performance. These findings highlight the importance of tuning the electrolyte concentration, composition, and thermal compatibility to optimize the intercalation behavior of layered materials in supercapacitor systems.

## Graphical abstract

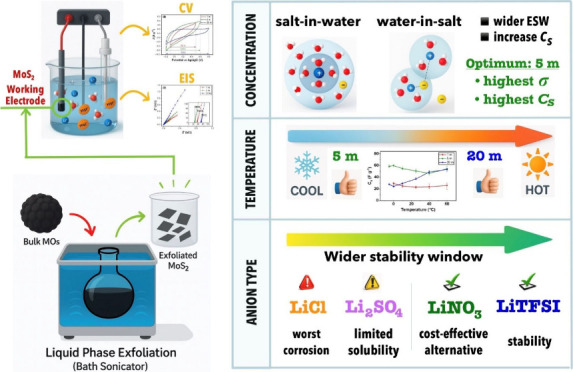

## Introduction

In recent years, improving energy density (stored energy) has become a central focus in the development of energy storage devices, such as supercapacitors and batteries. This is driven by the high energy demands required for developing storage cells suitable for electric vehicle applications.^1–3^ The key to achieving this goal lies in enhancing the intercalation performance of the electrode materials, which can be modified by controlling the interlayer spacing or by reducing the size of the intercalated ions within the material galleries.^4,5^ Hence, the exploration of two-dimensional (2D) layer materials, such as transition metal dichalcogenides (TMDs), and MXene,^6^ has broadened the landscape of the high intercalation degree of electrode materials^7^ apart from the metal oxide family.^8–10^

Specifically, transition metal dichalcogenides (TMDs) offer a compelling array of properties, such as tunable layer thickness and flake size.^11^ These features influence electrical conductivity, catalytic activity, and intercalation performance, significantly enhancing their electrochemical properties and providing a versatile platform for diverse applications.^12^ The electrochemical properties of TMDs are intrinsically linked to their structural dimensions, lateral size, and the number of stacking layers, with mono- or few-layer materials exhibiting distinct advantages,^13,14^ especially when performing liquid phase exfoliation and cascade centrifugation.^15^

Among the TMDs, molybdenum disulfide (MoS_2_) stands out as a promising electrode material.^16^ Its layered structure, analogous to graphene, facilitates ion intercalation and deintercalation, enabling the acquisition of pseudocapacitance alongside electric double-layer capacitance (EDLC).^17,18^ Cook et al.^19^ investigated the use of ordered mesoporous thin film MoS_2_ as a pseudocapacitive material for Li-ion and Na-ion charge storage. The synthesized material shows pseudocapacitive charge storage with higher specific capacitance beyond the traditional slow battery behavior, together with the fast charge–discharge of 20 s and durability of 10,000 cycles. Yoo et al.^20^ also investigated the intercalation pseudocapacitance of exfoliated MoS_2_ in nonaqueous Li-ion electrolytes. The exfoliated MoS_2_ exhibits significantly higher specific capacitance than previous reports with ultrafast kinetics, enabling high-rate capability for charge–discharge within 3 min. Therefore, understanding ion identities and electrolyte behavior^1^ becomes crucial in this context, especially as the hydrated ionic size is reduced when applying a super concentration (a so-called “water-in-salt”) electrolyte. A clear demonstration was provided by Suo et al., showing that water hydration shells become smaller with increasing LiTFSI concentration (up to 10 m), extending the conventional voltage of aqueous based electrolyte from 1.23 to 3.0 V.^21^ This is consistent with the report by Coustan and Bélanger, where superconcentrated LiTFSI electrolytes were shown to extend the capacitive voltage window up to 2.3–4.1 V.^22^ It is worth noting that high-concentration LiTFSI, known as a “water-in-salt” electrolyte, has been applied to layered MnO_2_ electrodes in both supercapacitor^23^ and battery^21^ applications to enhance energy density. This has drawn attention to the use of the original “water-in-salt” electrolyte (LiTFSI) as well as alternative high-solubility salts in the context of energy storage.^24^

Lithium-ion-based electrolytes, particularly those employing lithium bis­(trifluoromethanesulfonyl)­imide (LiTFSI), offer superior energy and power density, alongside a broad operating temperature range, compared to traditional aqueous electrolytes.^25^ LiTFSI stands out as a representative Li-ion electrolyte, exhibiting a wide electrochemical stability window, high ionic conductivity, and thermal stability.^26^ Pioneering work by Suo et al.^21^ has explored LiTFSI “water-in-salt” electrolytes for high-voltage lithium-ion batteries, and research has expanded to include other highly water-soluble lithium salts, such as lithium nitrate (LiNO_3_)^27^ and lithium chloride (LiCl).^28^ The observed widening of the electrochemical stability window in “water-in-salt” electrolytes is attributed to the altered solvation shell around ions, resulting from reduced water molecule availability, which effectively suppresses hydrogen and oxygen evolution reactions. Furthermore, those investigations have been conducted on Li-based salt electrolytes using activated carbon electrodes, where the charge storage behavior is primarily based on electric double-layer (EDL) mechanisms.^24^ In contrast, the understanding of intercalation processes and the ionic identities of LiTFSI and other high-solubility salts in “water-in-salt” systems remain underexplored.

This study investigates the electrochemical interaction between the layered materials, represented by using exfoliated MoS_2_, and “water-in-salt” electrolytes. The work explores the impact of varying electrolyte concentrations, transitioning from the traditional “salt-in-water” to “water-in-salt” system. To the best of our knowledge, there are no reports on the electrochemical behavior of anions at high concentrations in layered materials. Lithium-based salts, including LiTFSI, lithium nitrate (LiNO_3_), lithium sulfate (Li_2_SO_4_), and lithium chloride (LiCl)_,_ were investigated with a particular focus on the understanding of anion identities at high concentration. Furthermore, the study examines the influence of operating temperature ranging from −5 to 60 °C with LiTFSI, to simulate diverse environmental conditions for a practical use of “water-in-salt” electrolytes. This comprehensive analysis aims to provide valuable insights into electrolyte characteristics, contributing to the further development of high-energy-density devices.

## Results and Discussion

Overall, the exfoliated MoS_2_ was prepared via liquid-phase exfoliation using an n-propanol/water mixture as the exfoliation solvent (the material preparation/characterization can be found in Supporting Information sections S1 and S2). MoS_2_ was selected due to its widespread use in energy storage applications, including batteries^29,30^ and supercapacitors.^31–33^ It has a large interlayer spacing of 0.615 nm,^34–37^ which facilitates ion intercalation/deintercalation, serving as the model electrode for our studies. Moreover, the flake size and thickness can be precisely controlled using centrifugation techniques.^15^ This enables reproducible electrochemical results, offering a standardized understanding of the electrolyte characteristics. Figure 1A illustrates the comparative X-ray diffraction (XRD) patterns of bulk MoS_2_ (black line) and exfoliated MoS_2_ (red line). The peak corresponding to the (002) plane, indicative of interlayer spacing (*d*-spacing) between MoS_2_ layers, was observed at 2θ values of 14.4° and 14.5° for bulk and exfoliated MoS_2_, respectively. This is in good agreement with the previous literature.^18^ The broadened XRD pattern of exfoliated MoS_2_ signifies a decrease in crystallinity. This is attributable to the size reduction to smaller flakes, reduced in layer numbers, or nanosheets achieved through ultrasonication, alongside potential increases in defects, strain, disorder in planar or stacking configurations, and vacancies within the layers.^38,39^ The full XRD pattern is given in Figure S1. The surface chemistry of the exfoliated flakes was investigated using XPS, as illustrated in Figure 1B and Figure 1C for Mo 3d and S 2p species, respectively. The peak deconvolution information is provided in Table S1. Exfoliation led to a peak broadening without a shifting of binding energy. These phenomena suggest the consistent primary oxidation state of MoS_2_, but the electronic environment around these bonds is slightly altered.^40^ The increased full width at half-maximum (FWHM) values for all Mo and S in the exfoliated sample indicate disorder or heterogeneity, including edge sites, oxidation, defects, and the potential presence of an amorphous phase. This is typically observed when exfoliating the TMDs from bulk powder,^41–44^ which supports the XRD results. In addition to the structural and chemical properties, Figure 1D and Figure 1E illustrate SEM and TEM images of exfoliated MoS_2_. Obviously, the exfoliated flake exhibits a sheet-like structure, similar to other 2D materials. The flake size of exfoliated MoS_2_ is found to be around 100–300 nm with the thickness of 33.78 ± 3.75 nm (30.03–37.53 nm) and also the *d*-spacing which is relatively similar to those obtained from XRD. Based on comprehensive reviews of relevant literature,^45–48^ the characterization results of this study are comparable. Hence, it can be concluded that exfoliated MoS_2_ is a suitable material for electrochemical studies, serving as a representative model for other 2D materials.

**Figure 1:**
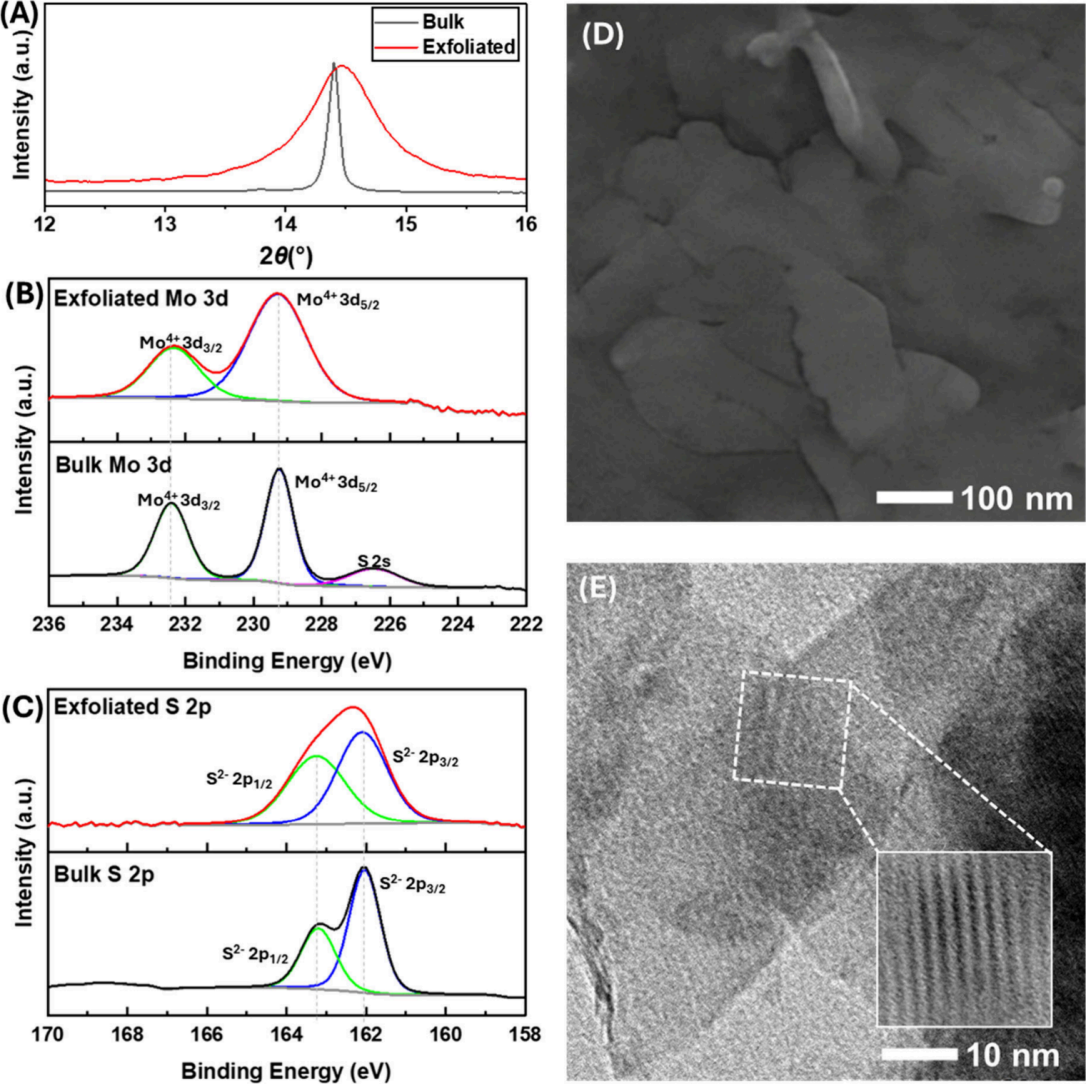
Morphology of bulk and exfoliated MoS_2_: (A) XRD patterns, (B) XPS spectra of Mo 3d, (C) XPS spectra of S 2p, (D) SEM image of an exfoliated MoS_2_ on a PVDF membrane, and (E) TEM images of exfoliated MoS_2._.

The electrochemical properties of MoS_2_ in various electrolytes were evaluated in a 3-electrode configuration. The evaluation procedure and the electrochemical stability window determination including the electrolyte preparation were mentioned in the Supporting Information. LiTFSI was initially selected in this study due to its well-established role as a “water-in-salt” electrolyte. The relationship for the concentration in molality [m] and molarity [M] is listed in Figure S2 and Table S4 with the electrolyte appearance in Figure S3. Figure 2A and B shows cyclic voltammetry of the electrochemical evaluation of different concentrations of LiTFSI with different scan rates at 1 and 100 mV s^–1^. Unlike carbon-based materials, where the voltage window of “water-in-salt” electrolytes is relatively wide (approximately 3 V),^24^ the MoS_2_ in 1 m LiTFSI electrolytes exhibit a much narrower window of around 0.6 V. This is due to the electrocatalytic effects including hydrogen (onset of −0.1 to −0.05 V vs RHE)^49,50^ and oxygen (overpotential +0.7 V vs RHE)^51^ evolution of the TMDs family. However, the LiTFSI electrolyte, with the concentration of 5 and 20 m, exhibits a widened voltage window of 0.8 and 1.0 V, respectively. Therefore, it is suggested that the reduction in water hydration with increasing salt concentration and reducing amount of free water^21^ can significantly suppress gas evolution, not only in carbon-based materials but also in TMD materials.

**Figure 2:**
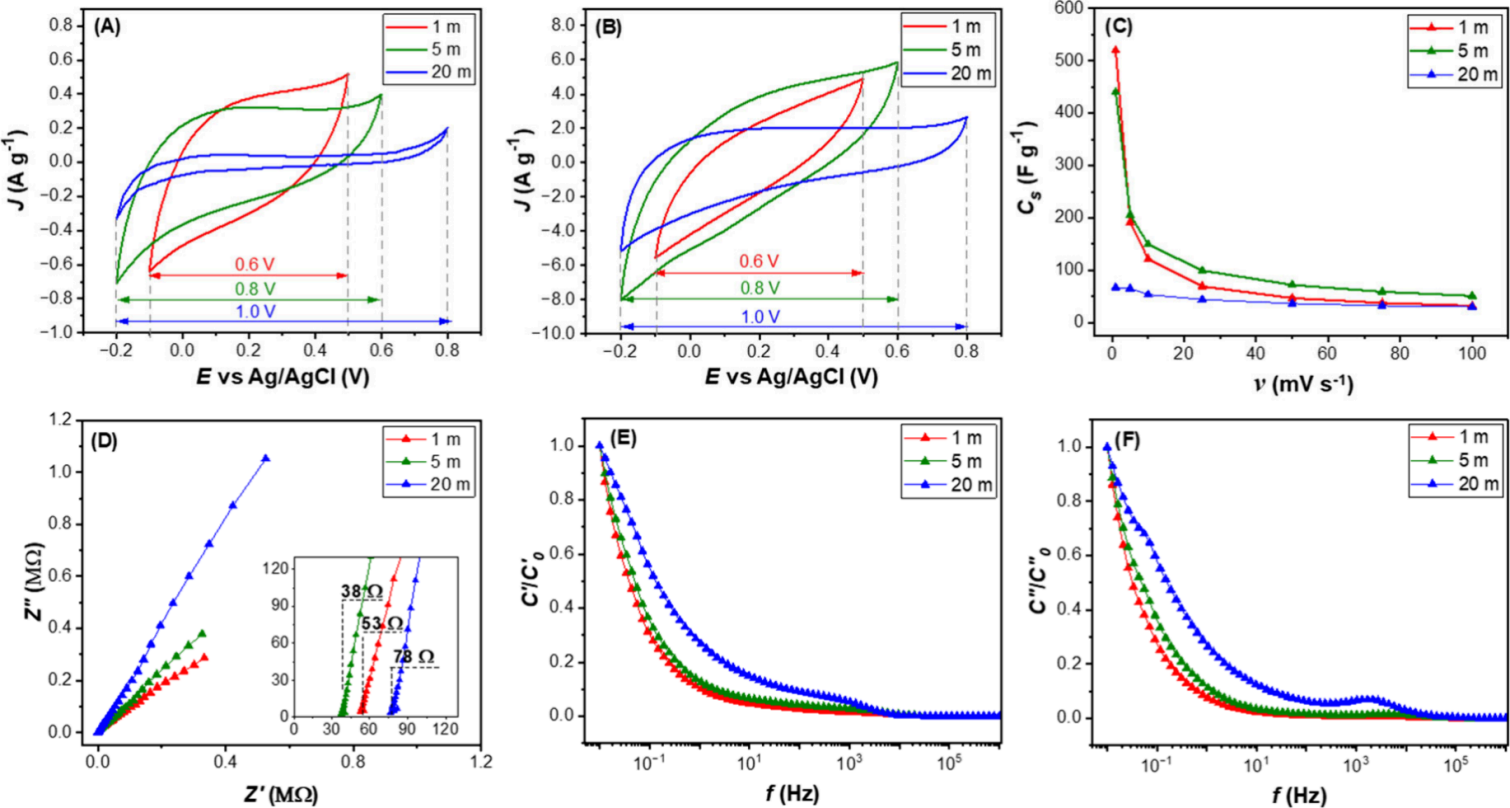
Electrochemical properties of MoS_2_ electrode in various LiTFSI electrolyte concentrations: (A) cyclic voltammetry of scan rate at 1 mV s^–1^ (low scan rate), (B) cyclic voltammetry of scan rate at 100 mV s^–1^ (high scan rate), (C) specific capacitance in different scan rates, (D) Nyquist plot, (E) Real capacitance of the system against frequency, and (F) imaginary capacitance of the system against frequency.

The specific capacitance variation along the different scan rates is illustrated in Figure 2C (The measured CVs are shown in Figure S7 with the numerical data in Table S6). The 1 and 5 m electrolyte demonstrate higher capacitance, when compared to the 20 m. This is due to its lower viscosity and high ionic conductivity. Conversely, the 20 m electrolyte exhibits the poorest capacitance (65 F g^–1^ at 5 mV s^–1^), which is attributed to its excessively high salt concentration leading to the ion pairing effect-impeded ion mobility; hence, it lowers the conductivity from 28.1 (at 1 m) to 10.0 mS cm^–1^. Note, the physiochemical properties of LiTFSI are illustrated in Figure S5 and Table S5. Yet, the 5 m electrolyte shows the highest capacitance up to 205 F g^–1^ at 5 mV s^–1^. This is due to the high ionic conductivity of 47.48 ± 0.11 mS cm^–1^ (the maximum value when tuning salt concentrations). The low concentration Li^+^ ions are surrounded be a well-defined primary solvation shell, with a secondary shell merging the ions into bulk water.^21^ The too bulky solvation shell, 1 m case, hinders ion mobility. In the case of 5 m, this concentration provides an optimal balance for ion transport and ensures nonlimited charge storage kinetics. The improved intercalation behavior observed with 5 m LiTFSI can be attributed to a partial desolvation of Li^+^, where the hydration shell is sufficiently reduced to permit interlayer diffusion without excessively hindering ion mobility. At this concentration, the number of water molecules per Li^+^ decreases from ∼55 (at 1 m) to ∼13, promoting more compact solvated ions capable of intercalating into MoS_2_ galleries. In contrast, the 1 m system features bulky solvation shells that restrict access, while the 20 m system suffers from excessive viscosity and ion pairing.

The solution resistance (*R*_*s*_) determined from the Nyquist plot, was found to be 53, 38, and 78 Ω for 1, 5, and 20 m of LiTFSI electrolytes, respectively (Figure 2D). The order of *R*_*s*_ is in good agreement with the ionic conductivity of the electrolyte, which is 5 < 1 < 20 m. It is obvious that the slope of the Nyquist plot becomes steeper when applied to high salt concentration, indicating more ideal capacitor behavior. The capacitance analyses, *C′* (Figure 2E) and *C′′* (Figure 2F), were also calculated from eqs S4 and S5, respectively. The frequency of 0.1–100 Hz is focused on as the transition zone of the electrochemical behavior shift from diffusion-controlled dominant to surface-controlled, reflecting the rate capability of the system. The real capacitance, *C′*, has a similar behavior for 1 and 5 m, but it is noticeably different for 20 m. This behavior implies the different charge storage mechanisms due to the difference between the solid/electrolyte interaction. The peak on *C′′* analysis reflects the longest relaxation time of the 20 m electrolyte (0.5 ms), which originates from the poor ion diffusion and charge. For the 1 and 5 m electrolytes, they provide faster relaxation times, 0.13 and 0.18 ms, respectively.

The effect of temperature on LiTFSI electrolytes at a variety of concentrations (1, 5, and 20 m) across a range of temperatures (from −5 to 60 °C) was studied. Figure 3A illustrates the temperature dependence of the MoS_2_/LiTFSI system via the CV curves. For all concentrations, the current response increases with increasing temperature from the faster ion mobility, reduced resistance, and then increased conductivity. A trend is consistent with the findings of Le Fevre et al., who studied the stability above 100 °C of the water-in-salt electrolytes.^52^ For each concentration, the CV curves maintained the same shape but changed in their enclosed area. This implies consistent charge storage mechanisms, although with varying amounts of charge storage. Therefore, changing the temperature is less effective for charge storage behavior. However, the electrochemical stability windows of the systems become narrower as the temperature increases, which is attributed to the faster activation of the water splitting reaction. The voltage required to kick-off the water splitting reaction in the electrolysis system becomes lower when the operating temperature increases.^53–55^ The same behavior was also found for the system with soluble ions as in the aqueous battery.^56^

**Figure 3:**
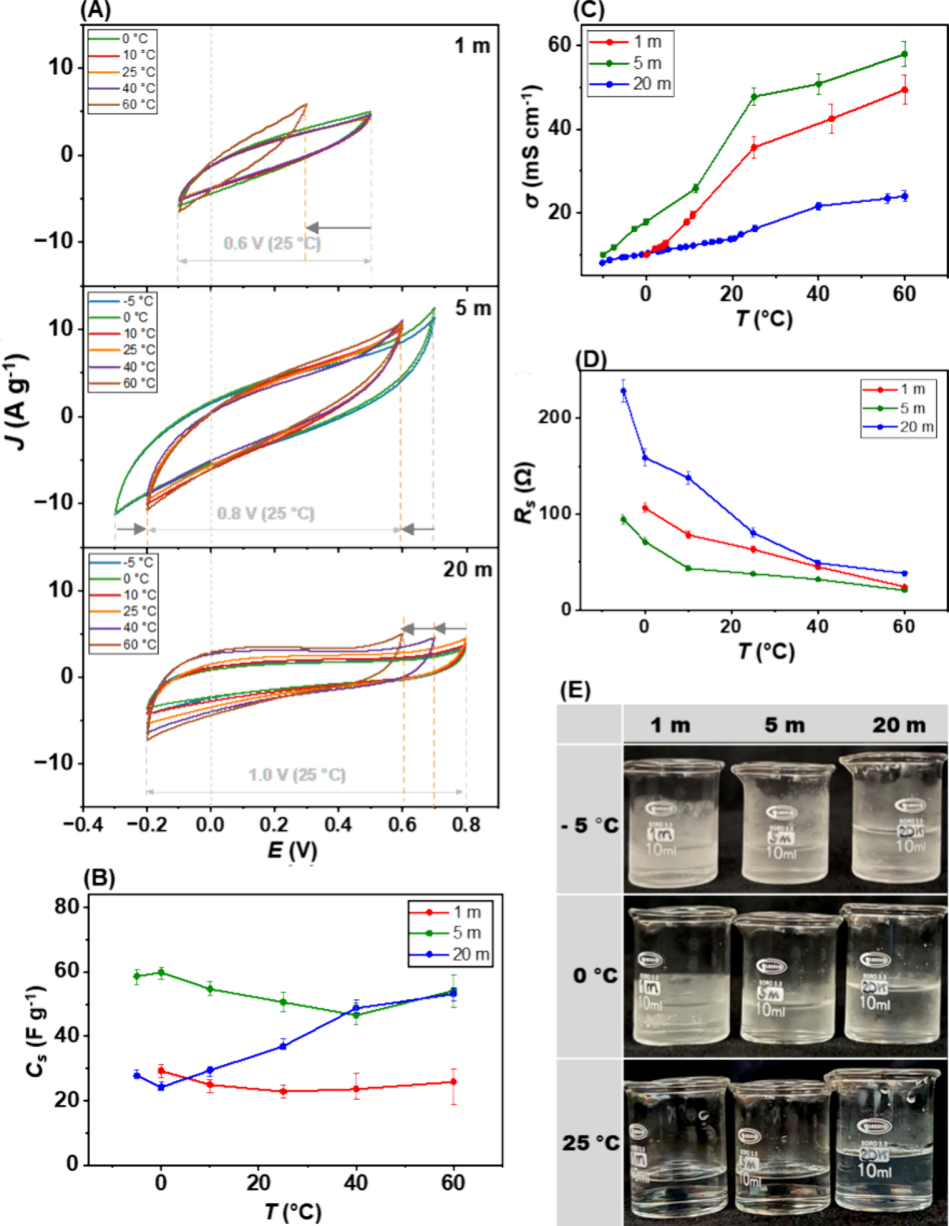
Electrochemical properties of MoS_2_ electrode in various LiTFSI electrolyte concentrations in a temperature dependent experiment: (A) cyclic voltammetry of different LiTFSI concentrations (scan rate at 100 mV s^–1^), (B) calculated specific capacitance at different operating temperatures, (C) electric conductivity of the electrolyte at different temperatures, (D) electrolyte solution resistance (R_s_) at different temperatures, (E) physical appearance of LiTFSI electrolyte at −5, 0, and 25 °C.

The 1 m electrolyte has a limited operational temperature range, performing consistently only between 0 °C up to 40 °C. Below 0 °C, measurements cannot be performed due to electrolyte freezing (Figure 3E) like pure water, which hinders ion mobility when it turns to its frozen state. At the upper end, after 40 °C, its stability window suddenly narrows to 60 °C (from 0.6 to 0.4 V). The current response of 1 m electrolyte from all temperature evaluations is lower, and the values of specific capacitance are also lower, compared to 5 and 20 m. This shows the relatively flat profile of 20–30 F g^–1^, implying less sensitivity toward the temperature changes. The relationship between temperature and specific capacitance (*C*_*s*_) is illustrated in Figure 3B, and the numerical data are listed in Table S8. The lowest *C*_*s*_ values throughout the temperature range are reasonable since the amount of charge is the lowest among the three electrolytes. The observation of water vaporization starts at 40 °C but clearly sees the change of electrolyte at 60 °C. At these elevated temperatures, the solvent loss causes an increased electrolyte concentration. At these elevated temperatures, solvent loss causes the electrolyte concentration to increase. Since both conductivity and solution resistance are highly dependent on concentration, this instability of the electrolyte leads to the fluctuation between measurement replications. While the solvation shell is essential for facilitating ion movement, the initial increase in concentration from evaporation can temporarily boost the ionic conductivity. However, excessive evaporation leads to a significant loss in specific capacitance, as was observed at 60 °C. Further heating causes the electrolyte to dry out, and the cell becomes inoperable and impractical for supercapacitor applications. These observations are consistent with the work of Zhang et al.^57^ They confirm the poor thermal stability of the 1 m electrolyte. The freezing near 0 °C is attributed to an unstable hydrogen-bond network from molecular dynamics (MD) calculations, and also the ice-like hydrogen-bond signal was found around 0 °C from Raman spectroscopy. Moreover, a noticeable structural transition occurs near 15–20 °C. Its bulk-like, water-rich composition allows a high ionic mobility in midrange temperature, but low and high-temperature instability limits its overall performance.

The 5 m electrolyte demonstrates an impressively wide electrochemical stability window of 1.0 V at −5 and 0 °C, achieving the highest specific capacitance of over 60 F g^–1^. At these temperatures, strong ion–solvent and ion–ion interactions disrupt the formation of a crystal lattice, allowing the electrolyte to remain liquid and preserve the ion mobility. The stability window narrows slightly to 0.8 V at higher temperature with a corresponding decrease in the specific capacitance. Its performance at low operating temperatures is beneficial for developing aqueous supercapacitors for cold climates. Furthermore, it delivers stable capacitance values (45–60 F g^–1^) with minimal variation, which is nearly twice the capacitance of the 1 m system. For the 20 m LiTFSI system, elevated temperatures lead to decreased viscosity and thermal disruption of ion pairs, effectively increasing the fraction of free mobile Li^+^. This temperature-driven reduction in interionic interactions facilitates faster ion transport and intercalation into MoS_2_, accounting for the significantly improved capacitance observed at 60 °C. Considering Figure 3C and Figure 3D, ionic conductivity and bulk solution resistance (determined from Nyquist plot, Figure S9) are inversely related; increasing temperature reduces viscosity by weakening intermolecular forces and also provides kinetic energy enhancing ion mobility and thus ionic conductivity. The findings go along well with the work by Zhang et al. Their study also identified the 5 m concentration to be the best overall performance. The excellent low-temperature behavior originates from a stable colloidal structure and a dynamic hydrogen-bond network resisting freezing, allowing the ion movement in the electrolyte. Their MD simulations further reveal a flexible Li^+^ hydration shell that adapts to the temperature changes. Aligning with our results, they also noted a slightly narrowed electrochemical stability window due to free water, also making the 5 m system to be less stable at high temperatures than the concentrated 20 m system.

The 20 m “water-in-salt” electrolytes display strong capacitive behavior, evidenced by more rectangular CV curves compared to the 1 and 5 m electrolytes. However, their performance is highly dependent on the temperature. At low temperature (−5 to 10 °C), the CVs become distorted with reduced current, but the electrolyte remains operationally stable, suggesting hindered ion transport from high viscosity. This is reflected in the solution resistance, as illustrated in Figure 3D, which varies with temperature. The highest value is over 200 Ω at subzero temperature but drops sharply to about 38 Ω at 60 °C. The specific capacitance is low at low temperatures but increases significantly with heating, approaching comparable values to 5 m at 60 °C. Besides this improvement, the electrolyte shows instability at 40 °C and above in this study, and its solution resistance remains higher than those of the 1 and 5 m electrolytes across all temperatures. The observations are well-aligned with the work of Zhang et al., as mentioned earlier for the 5 m electrolyte. Their research also confirms the best performance at high temperatures of the 20 m electrolyte, which is attributed to a highly stable structure where a tightly coordinated water within the Li^+^ solvation shells effectively suppresses side reactions. Furthermore, it is also confirmed that this structure leads to high viscosity, which hinders ion transport and causes poor rate performance and reduced capacitances at low temperatures, and even salting out. Therefore, the optimal performance of the MoS_2_/LiTFSI system depends on the electrolyte concentration and operating temperature. The 5 m electrolyte is best suited for low to moderate temperatures (−5 to 25 °C), while the 20 m electrolyte performs optimally at elevated temperatures above 40 °C. Notably, the 20 m electrolyte is highly responsive to temperature changes, exhibiting specific capacitance (*C*_*s*_) values comparable to 1 m at low temperatures and rising significantly with temperature to approach those of the 5 m system at higher temperatures, driven by enhanced ion dynamics.

The effect of ion identity from 1 m to maximum electrolyte concentrations is shown in Figure 4A; the CV curves were evaluated with a scan rate of 100 mV s^–1^. For 1 m electrolyte, electrochemical stability window was found to be 0.6 V with almost similar CV characteristics, while the CVs of maximum conditions exhibit a different *i*–*E* response. As expected, applying a higher salt concentration can extend the electrochemical stability window. The electrochemical stability window can be ranked as LiTFSI > LiNO_3_ > Li_2_SO_4_ = LiCl. Considering the anion sizes, solvation, and free water in the system, TFSI^–^ ions are very large with weak coordinating nature. These properties allow Li^+^ to be highly solvated with water molecules, resulting in reduced free water, a wider stability window, and higher energy density. Unlike a large anion like TFSI^–^, NO_3_^–^, SO_4_^2–^, and Cl^–^ are much smaller in size, and they provide their own intrinsic properties. The comparable candidate for 20 m LiTFSI is 20 m LiNO_3_. Even it shows a narrower electrochemical stability window of 0.8 V, compared to LiTFSI (1.0 V), but provides a comparable specific capacitance, especially in “water-in-salt” conditions (around 30 F g^–1^), as shown in Figure 4C. The smaller size of NO_3_^–^ itself and their solvate ions helps facilitate ion mobility through the electrode material, resulting in an improved specific capacitance. Moreover, LiNO_3_ is a more applicable option in applications due to its cost effectiveness.^27^ Li_2_SO_4_ exhibits the highest specific capacitance, compared to other types of Li salts, but it is noted that the concentration was limited at 3 m; therefore, it is not suitable to address it as a “water-in-salt” electrolyte. Its ion mobility is still facilitated due to its solvation structure and free water within the system. Therefore, the higher specific capacitance is mainly from the higher charge carrier density than the 1 m, which allows a densely packed EDL at the electrode surface.^58,59^ The conductivity and pH of each electrolyte can be found in Table S9 and Table S10. Lastly, LiCl exhibits the worst performance for both stability and specific capacitance. Chloride ions are relatively small in size, compared to the others, and also easy to be oxidized to chlorine gas.^60^ The corrosion of the electrode material can occur, leading to the narrowest electrochemical stability window and ineffective charge storage.

**Figure 4:**
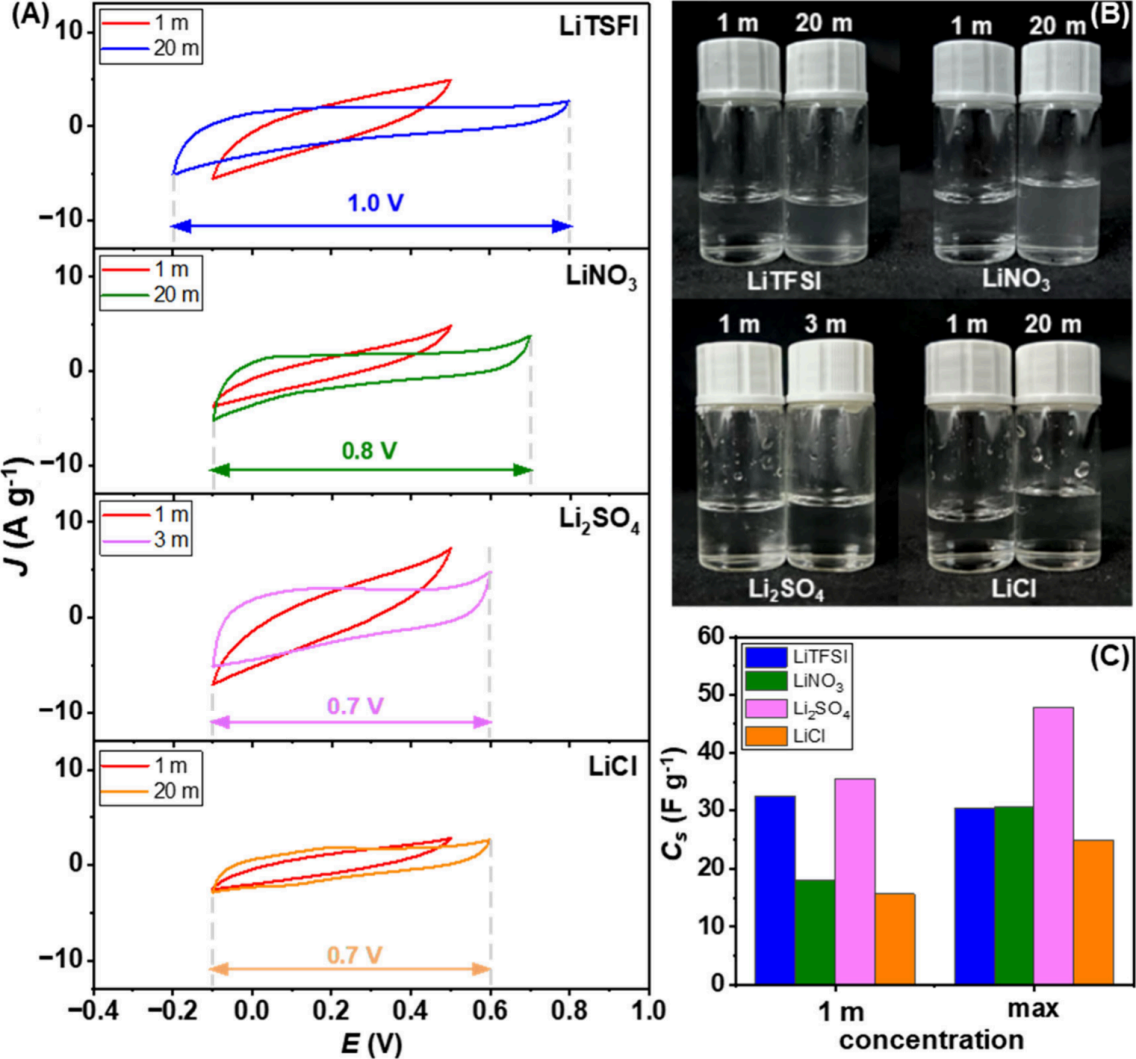
Electrochemical properties of MoS_2_ electrode in various Li-ion types of electrolytes: (A) cyclic voltammetry of different anion types with 1 m, and possible maximum concentration (scan rate at 100 mV s^–1^), (B) physical appearance of Li-ion electrolyte at room temperature, (C) calculated specific capacitance for different anion types of electrolytes.

In addition to CVs, EIS was employed, as illustrated in Figure S11 (1 m) and in Figure 5A (maximum concentration) with various types of anions. All salts exhibit a comparable solution resistance (*R*_*s*_) in the range of 16–25 Ω, except for LiTFSI (80 Ω). There are no distinct semicircles, suggesting very low charge transfer resistance. In contrast, a diffusion limitation becomes apparent in a low frequency region. In “water-in-salt” conditions, diffusion limitations greatly impact on mass transport of ions to/from the electrode and, consequently, the amount of charge storage. The steepest and longest line of LiCl indicates a severe diffusion limitation and the highest impedance among all salts. This hinders ion transport, resulting in the lowest specific capacitance. Li_2_SO_4_ shows a flatter and shortest line as the most diluted electrolyte with the lowest diffusion resistance and impedance, exhibiting the highest specific capacitance as a result. For LiNO_3_ and LiTFSI, the salts have comparable performance, even though the diffusion limitation in LiNO_3_ is higher from stronger ion pairing, and a slightly higher specific capacitance might be derived from its being smaller and more compact than the bulky TFSI anions, making LiNO_3_ superior in ion mobility.

**Figure 5:**
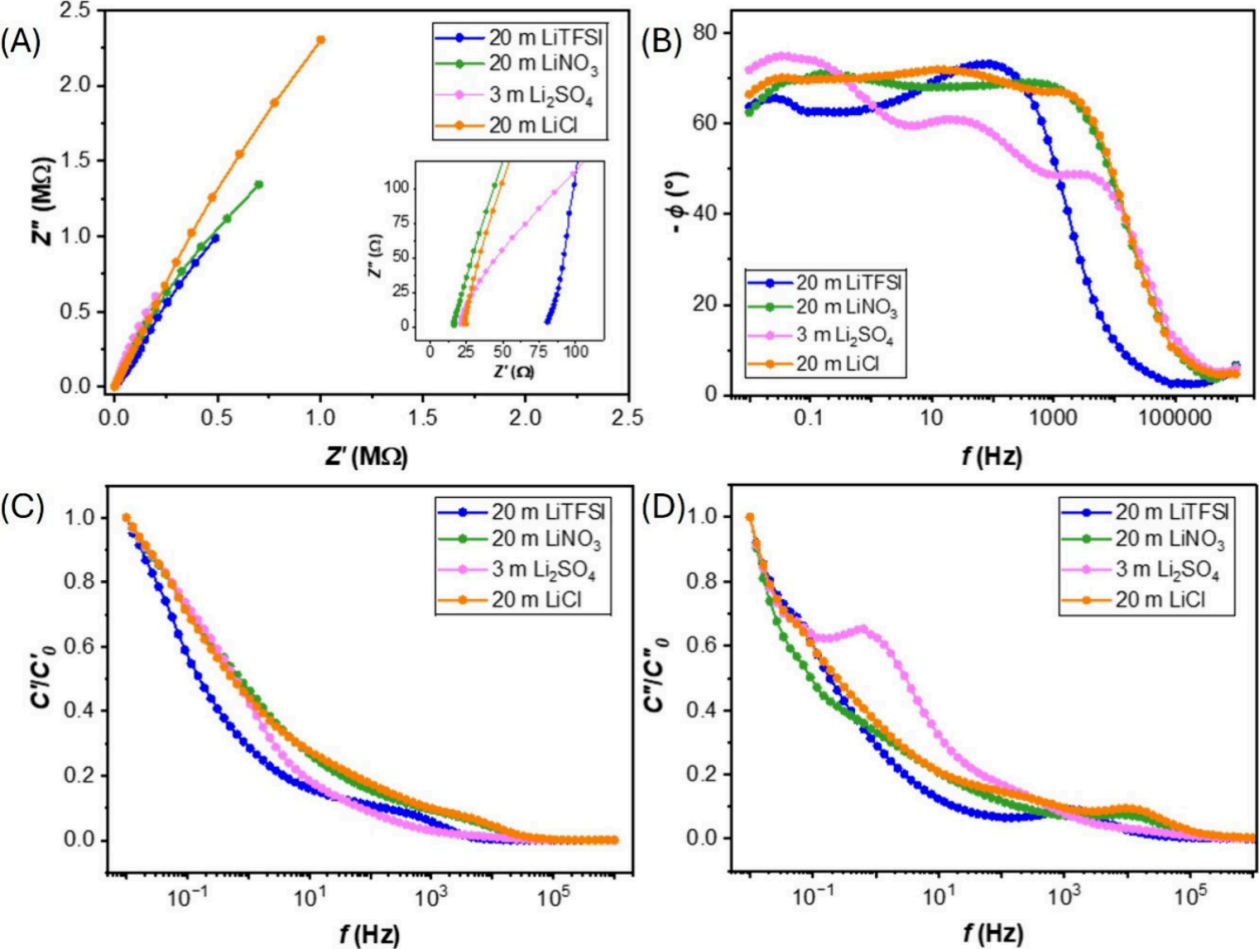
Nyquist plot (A), phase shift (B), and capacitance analysis (C and D) of the MoS_2_/Li-ion electrolyte system in various anion types of Li-ion electrolytes at the maximum possible concentration.

Figure 5B, C, and D illustrate the consideration of dynamic behavior (relaxation time), energy storage, and energy dissipation of the system. LiCl and LiNO_3_ show similar behavior. Their broad plateau frequency response at high phase angle across low to midfrequencies indicates the combination of capacitive-like and diffusion-controlled behavior with slow, distributed relaxation processes.^59,61^ This allows for charge storage capacity across various operating speeds but also implies a slow system response, which supports their results in the Nyquist plot. The observed slow decrease in charge storage and energy dissipation in slower rates over the frequency range indicates the challenges in ion movement.^58,59^ While these systems can store charges, their extremely low charged/discharged rates lead to continuous dissipation, resulting in a poor rate capability and inefficient energy storage. Li_2_SO_4_ shows a unique profile in frequency response. Its decreasing and fluctuating phase shift with increasing frequency suggests multiple relaxation processes with slightly varying time constants. A high phase angle at low frequencies indicates good capacitive behavior at slower rates. Its *C′* profile shows a faster decrease in capacitance in the midfrequency, compared to LiCl and LiNO_3_, indicating a worse rate capability. The distinct peak in *C′′* analysis at this frequency suggests that energy dissipation occurs at a specific rate. Lastly, LiTFSI exhibits the fastest relaxation, transitioning from capacitive to resistive behavior, evidenced by a phase shift peak in a midrange frequency (10–1000 Hz) followed by a sharp drop. Analysis of *C′* and *C′′* components confirms frequency-dependent performance, representing the reversible energy storage and irreversible energy dissipation. The system’s response at low frequencies is limited by slow diffusion-controlled ion transport. Once the frequency increases, the response is governed by a faster capacitive charging. However, excessively high frequencies cause a sharp drop in storage capability due to bulk resistance, limiting its high-rate applications.

## Conclusion

Exfoliated MoS_2_ was successfully prepared and served as a model TMD electrode to investigate the electrochemical performance of “water-in-salt”, a high-concentration aqueous electrolyte. LiTFSI was used to study the effect of electrolyte concentration and ion availability versus ion mobility. The 5 m LiTFSI electrolyte demonstrated the best overall performance at room temperature with the highest specific capacitance of 205 F g^–1^ (at 5 mV s^–1^), which was attributed to its peak ionic conductivity of 47.5 mS cm^–1^. In contrast, the 20 m “water-in-salt” electrolyte was limited by high viscosity and ion pairing, while the 1 m solution was limited by a lower charge carrier density. Temperature-dependent studies revealed distinct stability windows for each concentration. The 1 m electrolyte was unstable, freezing below 0 °C and degrading above 40 °C. The 5 m electrolyte showed excellent stability and high capacitance at low-to-moderate temperatures (−5 to 25 °C), whereas the 20 m electrolyte, despite poor performance at low temperatures, became optimal at elevated temperatures (above 40 °C) due to enhanced ion dynamics. Finally, comparisons across various Li-ion electrolytes confirmed the critical role of anion identity. LiTFSI was highlighted for the widest stability window (1.0 V) with the fastest relaxation dynamics. LiNO_3_ emerged as a promising, cost-effective “water-in-salt” alternative with a comparable specific capacitance to LiTFSI. Although 3 m Li_2_SO_4_ delivered a high specific capacitance, its limited solubility prevents its classification as a “water-in-salt” system. LiCl exhibits the poorest performance, attributed to its narrow stability window and the potential for corrosive side reactions. These findings highlight the importance of tuning both electrolyte concentrations and ion identity to optimize the supercapacitor performance for specific operational conditions.

## Supplementary Material



## Notes

Supplementary data for calculations and detailed explanations, including the preparation of MoS_2_ via liquid-phase exfoliation, a detailed characterization by XRD and XPS, preparation of electrolytes, the physiochemical properties of Li-ion electrolytes, electrochemical evaluation in a 3-electrode system for MoS_2_/LiTFSI in various electrolyte concentrations, different operating temperatures, and variations of Li-anions (in each section including determination of the electrochemical stability window, determination of specific capacitance from CV, EIS evaluation, and capacitance analysis). (PDF)
