# Supplementary material for: Understanding Ion Intercalation Characteristics of Layered Materials in Superconcentrated Electrolytes: Effects of Concentration, Temperature, and Anion Identity
Source: ACS Omega. 2025 Sep 4;10(36):41938–47. doi: 10.1021/acsomega.5c06741 (PMC12444522; doi:10.1021/acsomega.5c06741)
Supplement: Supplementary file 1 [file ao5c06741_si_001.pdf]

SUPPORTING INFORMATION

Understanding Ion Intercalation Characteristics of Layered Materials in Superconcentrated Electrolytes: Effects of Concentration, Temperature, and Anion Identity

Sirintra Arayawate[a,b], *Napaporn Kareeklin*[a,b], *Thanit Saisopa*[c], *Prayoon Songsiriritthigul*[d], *Pawin Iamprasertkun*[a,b]*

[a] School of Bio-Chemical Engineering and Technology, Sirindhorn International Institute of Technology, Thammasat University, Thailand (pawin@siit.tu.ac.th)

[b] Research Unit in Sustainable Electrochemical Intelligent, Thammasat University, Thailand

[c] Department of Applied Physics, Rajamangala University of Technology Isan, Thailand

[d] School of Physics, Suranaree University of Technology, Nakhon Ratchasima, 30000, Thailand

E-mail: pawin@siit.tu.ac.th (P. Iamprasertkun)

Keywords: TMDs, MoS_2, “water-in-salt”, Temperature, Ions

Table of contents

S1. Preparation of MoS_2 electrode materials 3
S2. Characterization of exfoliated MoS_2 3
S3. Preparation of electrolytes 5
S4. Properties of LiTFSI Electrolytes 8
S5. Electrochemical properties evaluation of MoS_2-LiTFSI in 3-electrode system 12
- Determination of electrochemical stability window (ESW) of MoS_2 in various LiTFSI concentration 12
- Determination of specific capacitance and capacitance analysis 13
- Calculation and interpretation of capacitance analysis (*C'* and *C"*) 14
S6. Performance evaluation in different operating temperatures 15
- Determination of ESW in temperature variation experiments 15
- Determination of specific capacitance in temperature variation experiment 17
- EIS evaluation in temperature variation experiments 17
S7. Performance evaluation in different variations of Li anions 19
- Determination of ESW in anion variation experiment 20
- Determination of specific capacitance in anion variation experiment 21
- EIS evaluation in anion variation experiments 22
References 23

S1. Preparation of MoS_2 electrode materials

Molybdenum(IV) sulfide (MoS_2) powder in bulk form from Sigma Aldrich, USA was used with the mixture of solvents, consisting of Isopropyl alcohol (Propan-2-ol, ≥ 99.8% AR grade, Fisher Scientific, USA) and in-house deionized water (measured resistivity at 25 °C is 17 MΩ.cm) with 50:50 volume ratio of batch size 200 mL with the concentration of MoS_2 of 10 mg mL^{-1}. The sample was placed in a bath sonicator. The bath sonication was performed while maintaining bath temperature at 20 – 25 °C continuously for 12 hours in a round bottom flask at intensity of 3 of the bath sonicator (max power 360 W, 45 kHz, operating at 13.3% of max amplitude which is equivalent to 192 W). After the completion of sonication, the dispersed sample was centrifuged at 5000 rpm (4109 g) for 20 minutes for 2 times to surely remove the unexfoliated MoS_2 as the sediment. The supernatant was collected as exfoliated MoS_2 and further used in this study. The collected sample was redispersed in the bath sonicator before drop casting on the glassy carbon working electrode every time.

S2. Characterization of exfoliated MoS_2

All of the characterizations were evaluated in comparison between bulk and exfoliated MoS_2. X-ray diffraction (XRD, Bruker AXS D8 Advance, Germany) with Cu $K\alpha_1$ (wavelength of 1.5406 Å) radiation at voltage of 40 kV, current at 40 mA. The evaluation angle (2θ) is 5 – 80 degrees with 0.02 step size and 0.2 step time using LYNXEYE XE-T detector. The full spectra are illustrated in Figure S1. The peak position of (002) plane of MoS_2 was used to calculate the d-spacing of the material using equation S1, Bragg's law.

$$d = \frac{\lambda}{2sin\theta} \tag{S1}$$

where λ is the wavelength of the radiation source, 1.5406 Å, and θ is a half value of the (002) peak position obtained in XRD pattern.

Utilizing Bragg's law, equation (S1), the calculated d-spacing was approximatly 6.15 Å for bulk, and exfoliated MoS_2. As a bulk solid, TMDs hold each other with van der Waals force. Once it was exfoliated, the influence of force in the environment was also changed.[1]

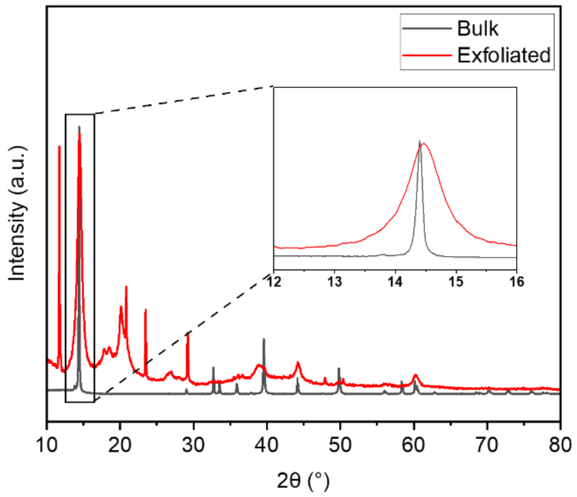

Figure S1. Full-range spectra of XRD measurement of MoS_2.

X-ray photoelectron spectroscopy (XPS) acquired with XPS spectrometer using a monochromate Al Kα_1 X-ray source operated at 1.56 A and 15 kV. Binding energy of Mo 3$d_{3/2}$ 3$d_{5/2}$ and S 2$p_{1/2}$ 2$p_{3/2}$ of bulk and exfoliated MoS_2 were compared in order to confirm the oxidation states of the materials. The spectra of Mo and S were shown in Figure 1 in the main text. The detailed calculations of the area, area ratio, doublet ratio, and FWHM are listed in Table S1 below.

Table S1. Area ratio and FWHM determined in XPS spectra.

**Parameter**		**Mo**		**S**	
		**Mo 3$d_{3/2}$**	**Mo 3$d_{5/2}$**	**S 2$p_{1/2}$**	**S 2$p_{3/2}$**
Binding Energy (eV)	Bulk	232.4	229.24	163.20	162.03
	Exfoliated	232.34	229.29	163.25	162.1
	Shifting	- 0.06	+ 0.05	+ 0.05	+ 0.07
Area (abs)<br>Area (%)<br>Doublet ratio	Bulk	4142.34<br>(44.20%)<br>2	5229.33<br>(55.80%)<br>2.52	1220.43<br>(35.32%)<br>1	2234.97<br>(64.68%)<br>1.83
	Exfoliated	1262.37<br>(29.26%)<br>2	3052.14<br>(70.74%)<br>4.84	1126.07<br>(46.65%)<br>1	1287.74<br>(53.35%)<br>1.14
FWHM	Bulk	1.16	0.96	1.02	0.93
	Exfoliated	1.75	2	1.73	1.48

The significant changes in doublet ratios are observed, where Mo $3d_{5/2}$ contribution increased from 55.80% in the bulk to 70.74% in the exfoliated material. It further supports the alteration of the electronic environment around Mo and S atoms, specifically, a marginal increase in electron density around Mo and a decrease around S in the exfoliated sample. These alterations may stem from modified interlayer interactions due to size reduction, the presence of defects or vacancies, surface modifications, or interactions with adsorbates or the IPA/water substrate used during exfoliation.

The morphology of the exfoliated MoS_2 electrode, including surface morphology and flake size, were illustrated using field-emission scanning electron microscope (FE-SEM; JEOL JSM7800F, JAPAN) with an accelerated voltage at 2 – 15 kV, and also a transmission electron microscope (TEM; JEOL JEM-2100 Plus, JAPAN) with accelerated voltage of 200 kV. The SEM and TEM images were illustrated in Figure 1D and 1E in the main text, respectively.

S3. Preparation of electrolytes

Li-ion electrolytes were prepared by molality (mol kg^{-1}) with the concentration listed as follows: LiTFSI (1 – 20 m), $LiNO_3$ (1 and 20 m), Li_2SO_4 (1 m and 3 m), and LiCl (1 and 20 m), respectively. The data of solubility of each salt is shown in Table S2 as their maximum solubility. LiTFSI was prepared in many concentrations to illustrate their solution properties as a typical Li-ion electrolyte. For the rest, 1 m, which is representative of salt-in-water electrolyte, and the maximum concentration, without recrystallization of salts within the limit of solubility as listed in

Table **S*3***, were prepared as the representative of water-in-salt electrolytes .

Table S2. List of Li salts used with purity and its origin.

**Li salts**	**Company**
Lithium bis(trifiuoromethane-sulfonyl) imide (LiTFSI)	TOB NEW ENERGY, China
Lithium nitrate ($LiNO_3$, 99.9% purity)	Acros Organics, Belgium
Lithium sulfate (Li_2SO_4, 99.9% purity)	Acros Organics, Belgium
Lithium chloride (LiCl, 98% purity)	Kemaus, Australia

Table S3. The solubility of LiTFSI, $LiNO_3$, Li_2SO_4, LiCl and LiTFSI electrolytes [2].

Electrolytes	Solubility
**LiTFSI**	602.89 /100 mL (20 °C) //21.00 m
**$LiNO_3$**	172.36 g/100 mL (25 °C) //25.00 m
**Li_2SO_4**	34.08 g/100 mL (25 °C) //3.10 m
**LiCl**	102.17 g/100 mL (25 °C) //24.10 m

For the ease of understanding of the typical LiTFSI electrolyte, the electrolyte concentration in this study is solely mentioned as molality. These are the numbers of moles of Li-ion vs the numbers of moles of water which reflect the interaction of Li-ion and water molecules, i.e., how the Li-ions were surrounded by water molecules. These phenomena are considered as solvation shell, which are the factors that affect the ions movement of the electrolytes and reflect the electrochemical performance of the system. The common term for solution concentration is molarity which is the numbers of moles of Li-ion per volume in liter of a solution. Here, the molarity of LiTFSI is also revealed in Figure S2, and numerically listed in Table S4.

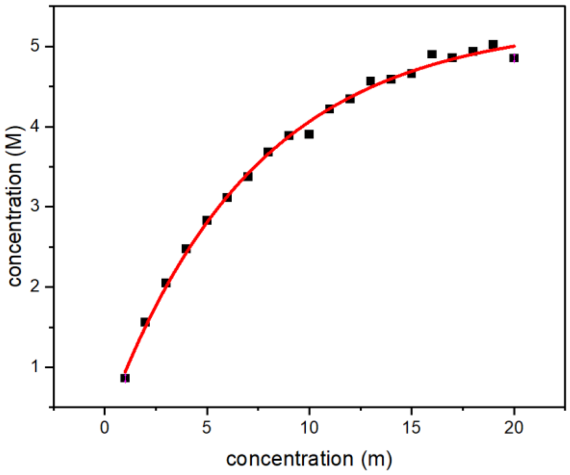

Figure S2. The relationship between the concentration in molarity and molality.

Table S4. The LiTFSI concentration in molality and molarity used in this study.

Calculated molality (m)	Experiment molality (m)	Experiment Molarity (M)
1	1.00	0.8618
2	2.00	1.5626
3	3.00	2.0516
4	3.99	2.4722
5	5.00	2.8271
6	5.99	3.1115
7	7.00	3.3715
8	7.99	3.6843
9	9.00	3.8853
10	10.00	3.9028
11	10.97	4.2185
12	11.98	4.3470
13	12.99	4.5668
14	13.96	4.5919
15	14.99	4.6616
16	16.00	4.9016
17	16.98	4.8554
18	17.89	4.9380
19	18.97	5.0228
20	19.98	4.8536

S4. Properties of LiTFSI Electrolytes

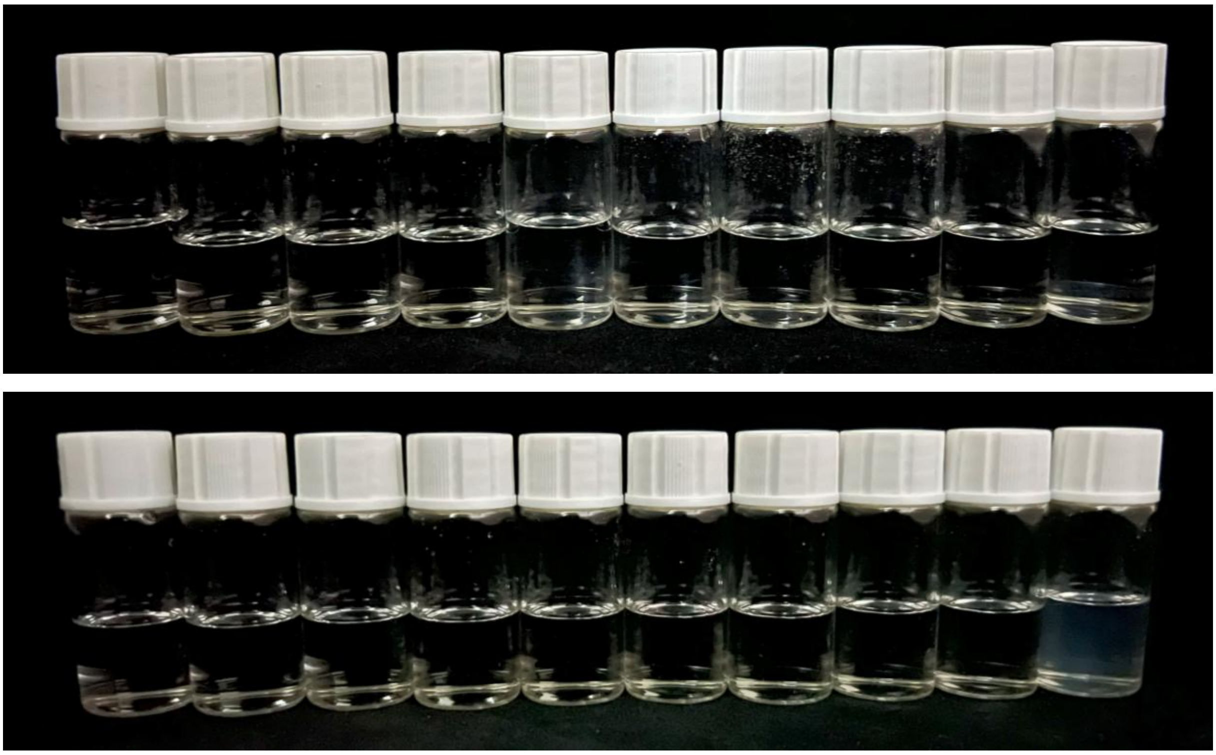

Figure S3. LiTFSI appearance and turbidity of prepared LiTFSI electrolytes from 1 – 10 m (top) and 11 – 20 m (bottom).

The evaluation of electrochemical properties of MoS_2 are determined at different concentrations from salt-in-water to water-in-salt LiTFSI electrolyte. The physiochemical properties of the prepared electrolytes, including electrolyte density with the relationship to the calculated Li:H_2O molar ratio in the prepared electrolytes, calculated dynamic viscosity obtained from the measured kinematic viscosity, conductivity, and pH value.

The kinematic viscosity (ν, mm^2/s) of LiTFSI electrolytes was measured using Ubbelohde Viscometer Size 1 (ISO/TR 3666, 0.17%, Cannon Instrument Company) with a constant = 0.009930 mm^2/s^2. The kinematic viscosity data calculated using equation S2 was illustrated in Figure S4. This data was further converted to dynamic viscosity (η) using equation S3 and illustrated in Figure S5(B). The pH value of the electrolytes and conductivity, measured by a pH and conductivity meter (Mettler Toledo, Greifensee, Switzerland), were shown in Figure S5 (C) and (D).

$$\nu = constant \times t \qquad \text{(S2)}$$

$$\nu = \frac{\eta}{\rho} \qquad \text{(S3)}$$

where, v is Kinematic viscosity, η is dynamic viscosity and ρ is a measured density.

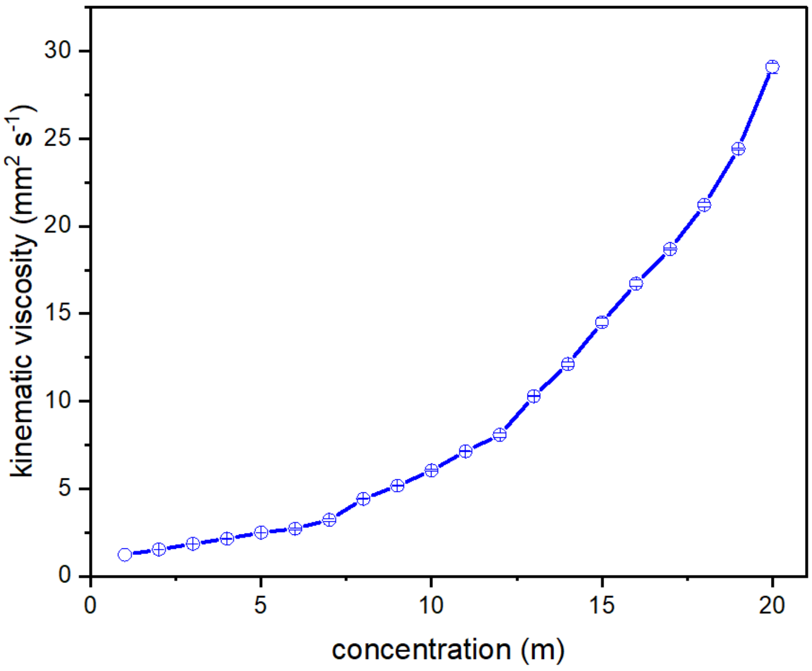

Figure S4. Kinematic viscosity of LiTFSI electrolytes in various concentrations.

Table S5. Numerical data on the properties of prepared LiTFSI electrolytes.

**Concentration (m)**	**Density (g/mL)**	**Kinematic viscosity (cSt)**	**Dynamic Viscosity (cP)**	**Ionic conductivity (mS/cm)**	**pH**
1	1.11	1.22 ± 0.00	1.35 ± 0.00	28.13 ± 0.06	7.22 ± 0.03
2	1.23	1.53 ± 0.02	1.88 ± 0.02	40.80 ± 0.08	7.26 ± 0.04
3	1.27	1.85 ± 0.01	2.36 ± 0.01	45.67 ± 0.19	7.36 ± 0.03
4	1.33	2.14 ± 0.02	2.85 ± 0.02	47.13 ± 0.08	7.41 ± 0.04
5	1.38	2.49 ± 0.02	3.43 ± 0.02	47.48 ± 0.11	7.56 ± 0.03
6	1.41	2.71 ± 0.05	3.83 ± 0.07	46.57 ± 0.07	7.44 ± 0.02
7	1.45	3.22 ± 0.07	4.67 ± 0.10	44.57 ± 0.18	7.21 ± 0.02
8	1.52	4.42 ± 0.02	6.71 ± 0.02	39.26 ± 0.06	7.12 ± 0.03
9	1.55	5.17 ± 0.04	8.00 ± 0.06	35.74 ± 0.26	6.96 ± 0.02
10	1.51	6.04 ± 0.06	9.13 ± 0.09	32.39 ± 0.19	6.90 ± 0.04
11	1.60	7.14 ± 0.03	11.40 ± 0.05	28.61 ± 0.02	6.84 ± 0.04
12	1.61	8.08 ± 0.12	13.01 ± 0.20	25.48 ± 0.06	6.83 ± 0.09
13	1.66	10.28 ± 0.03	17.09 ± 0.05	22.42 ± 0.12	6.77 ± 0.05
14	1.65	12.11 ± 0.14	19.96 ± 0.23	19.94 ± 0.04	6.72 ± 0.03
15	1.65	14.50 ± 0.21	23.92 ± 0.34	17.26 ± 0.02	6.63 ± 0.04
16	1.71	16.72 ± 0.20	28.64 ± 0.35	15.22 ± 0.04	6.40 ± 0.01
17	1.68	18.69 ± 0.08	31.40 ± 0.13	13.48 ± 0.04	6.29 ± 0.04
18	1.69	21.21 ± 0.12	35.92 ± 0.20	11.98 ± 0.01	6.21 ± 0.07
19	1.71	24.42 ± 0.05	41.69 ± 0.08	10.46 ± 0.06	6.27 ± 0.06
20	1.64	29.11 ± 0.34	47.64 ± 0.56	10.05 ± 0.01	6.26 ± 0.01

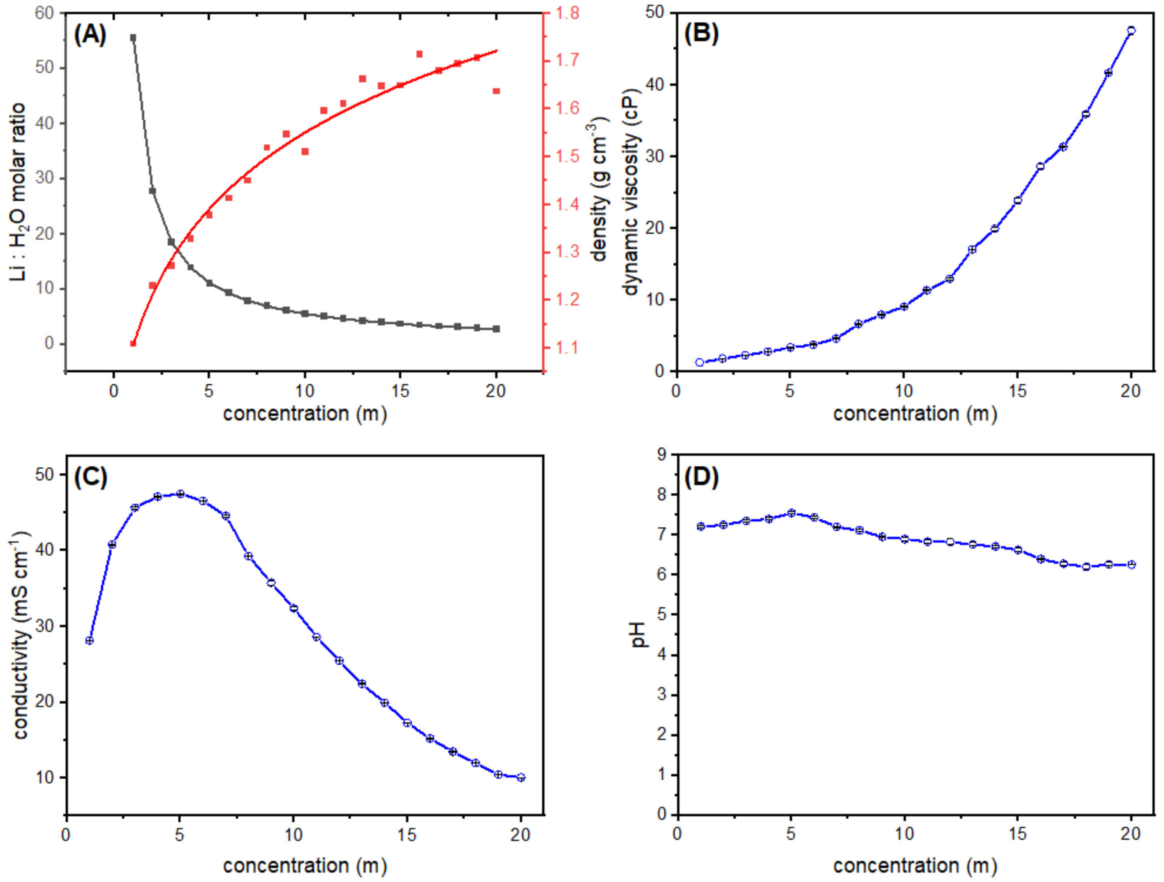

Figure S5. Physicochemical properties of LiTFSI electrolyte in different concentrations; (A) Li:H_2O ratio and density, (B) dynamic viscosity, (C) conductivity, and (D) pH.

LiTFSI electrolyte solutions were prepared in the molality of 1 – 20 m which is the concentration variation from “salt-in-water” to “water-in-salt” condition. The physiochemical properties of these electrolytes were illustrated in Figure S5. The amount of LiTFSI in the electrolytes impacts the properties of the solution. The higher concentration of LiTFSI increases in both the density and viscosity of the electrolytes. However, the molar ratio of water molecules surrounding Li-ion is notable decrease, especially from 1 – 5 m in which the water molecules decrease from 55 to 13 molecules. This trend continues with the slower rate from 5 m onwards, until it reaches 3 molecules at 20 m.

The number of water molecules surrounding Li-ion, i.e., the size of solvation shell, directly affects the physiochemical properties of the electrolyte. Too large solvation shell hinders the mobility of Li ions. On the other hand, a small solvation shell leads to a stronger interaction between water molecules and Li ions and alters the interaction and mobility of ions in the electrolytes. This suggestion is supported by the electrolyte conductivity as presented in

Figure S5(C). From the figure, it suggests that the optimum size of solvation shell will allow the high ion movement. The phenomenon is found at the concentration of 5 m which provides the highest conductivity. The pH of the electrolytes is another property which relates to ion movement and also relates to hydrogen activity which is related to water activity in the electrolyte. The pH values of all LiTFSI electrolytes remain relatively stable in a range of 6.2 to 7.5, which are considered as neutral electrolytes, as shown in Figure S5(D). According to these observations, the selected concentration of LiTFSI is 1, 5, and 20 m as representatives of salt-in-water, highest conductivity, and water-in-salt electrolyte, respectively. In the main text, these electrolytes will be used in the electrochemical performance evaluation in the first part of the discussion.

S5. Electrochemical properties evaluation of MoS_2-LiTFSI in 3-electrode system

The glassy carbon working electrode of three-electrode testing was prepared by drop-casting of the exfoliated MoS_2 onto it. The same active mass of exfoliated MoS_2 was prepared for all evaluations. The polycrystalline Pt wire was used as counter electrode and double junction Ag/AgCl electrode was used as reference electrode. The evaluation was conducted in cyclic voltammetry for ESW, determination of specific capacity vs scan rate, EIS for impedance and capacitance analysis using potentiostat (Palmsens4, the Netherlands)

Determination of electrochemical stability window (ESW) of MoS_2 in various LiTFSI concentration

To determine the electrochemical stability window of the system, there are several criteria for consideration. In this study, the cyclic voltammetry from zero potential measuring in positive potential and negative potential were done separately with the potential increment/decrement steps of 0.1 V towards those sides. The voltage that results in obvious reduction or oxidation peak in CV curves are considered as a maximum applied voltage for each side. The potential range before the reaction occurs, i.e. change in current response, are considered as ESW for this study. The CV measurement results (at a scan rate of 100 mV s^{-1}) and ESW of the system with different LiTFSI electrolyte concentrations are illustrated in

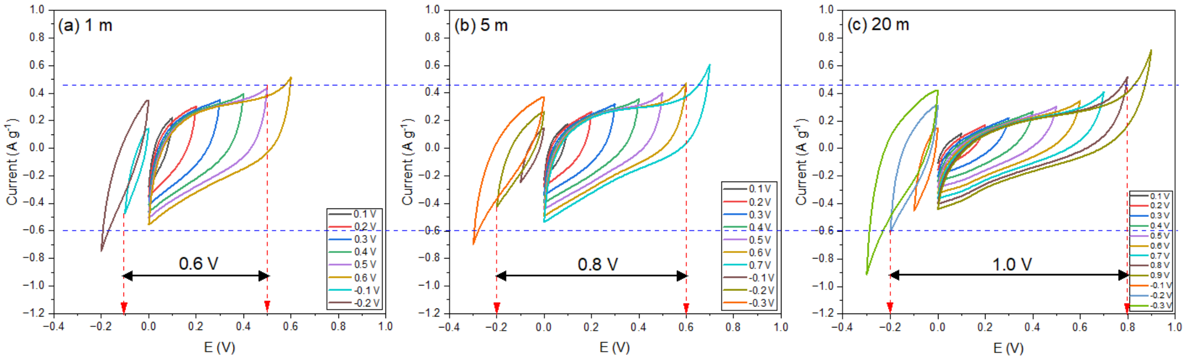

Figure **S6**. By considering the same current response for both negative and positive potential, the ESW can be selected as follows: 1 m (from -0.1 to 0.5, ESW = 0.6 V), 5 m (from -0.2 to 0.6, ESW = 0.8 V), and 20 m (from -0.2 to 0.8, ESW = 1.0 V). These ESW are considered in section 1 of the results and discussion. The increasing of salt content in an electrolyte clearly widens the electrochemical stability window of the system but the current response on the positive side becomes lower while comparing at the same positive voltage. This implies the lower possibility of oxidation reaction, preference towards reduction of the electrode material, diffusion limits of the ions in anodic sweep, etc.

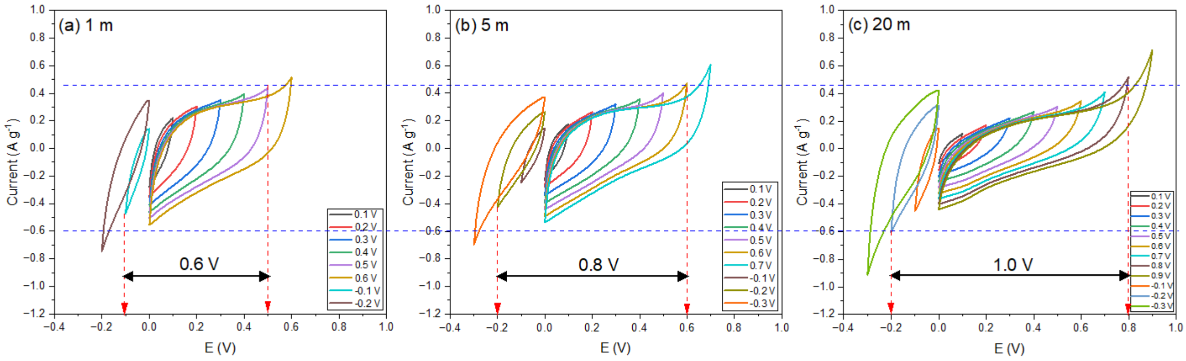

Figure S6. Selecting the working potential window.

<u>Determination of specific capacitance and capacitance analysis</u>

In order to study charge transfer mechanisms of supercapacitors, detailed calculations of capacitance are indispensable. In this part, the calculation of specific capacitance in different scan rates are discussed, together with the capacitance towards frequency response from EIS measurements. Specific capacitance calculation (Table S6) from CV measurements from different scan rates (Figure S7) reflect the intercalation and conversion mechanisms. A high scan rate provides inadequate time for ions to diffuse into electrode material. In order to

analyze the phenomena, the calculation of capacitance via CV curves in this study was calculated based on the following equation:

$$C_{CV} = \int \frac{I\,dV}{m\,\upsilon\,\Delta V} \qquad \text{(S4)}$$

where $\int IdV$ is the area of the CV closed curve (A g^{-1} V), m is the mass of active material casted on the electrode (g), υ is the electrode potential scan rate (V s^{-1}), and ΔV is the potential window (V).

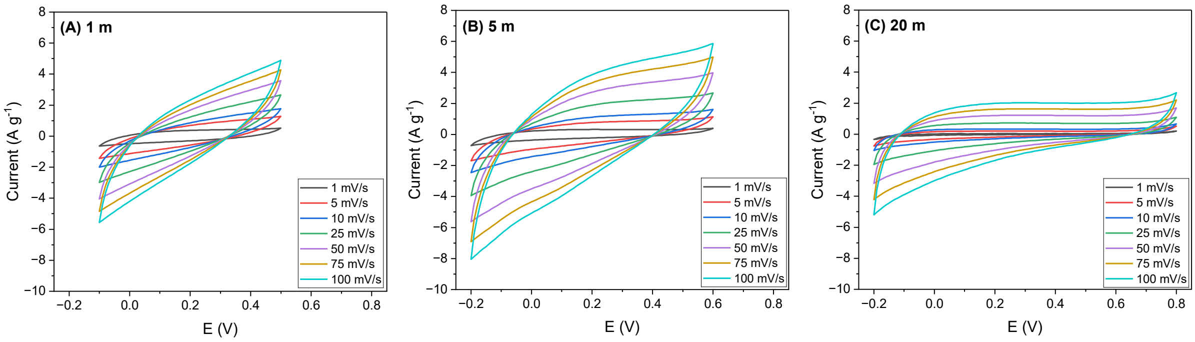

Figure

Table S6. Numerical data of area integration and C_s value calculation.

**Scan rate (V s^{-1})**	**C_S (F g^{-1})**		
	**1 m**	**5 m**	**20 m**
1	519.4	439.7	67.4
5	191.8	206.0	65.5
10	121.7	149.6	53.9
25	69.3	99.8	44.4
.50	47.1	72.3	37.0
75	37.7	59.1	32.8
100	32.6	51.4	30.4

<u>Calculation and interpretation of capacitance analysis (*C'* and *C''*)</u>

Besides CVs, the data from electrochemical impedance spectroscopy (EIS) also informs the charge storage mechanism. In this study, EIS was performed at 0.0 V using an amplitude of 10 mV perturbation in the frequency range of 0.01 - 100 kHz. The real and complex capacitance components were calculated according to Equation (C[illegible](ω)[illegible]$'$[illegible] $=$

$\frac{-Z''_{(\omega)}}{\omega\,|Z_{(\omega)}|^2}$ (S5) and (C[illegible](ω)[illegible]"[illegible] $= \frac{Z'_{(\omega)}}{\omega\,|Z_{(\omega)}|^2}$ (S6) from the impedance data. The calculation of capacitance as a function of frequency helps us to understand the frequency response of the MoS_2-LiTFSI system with different LiTFSI concentrations, illustrated in Figure 2E and 2F (in main text).

$$C'_{(\omega)} = \frac{-Z''_{(\omega)}}{\omega\,|Z_{(\omega)}|^2} \qquad \text{(S5)}$$

$$C''_{(\omega)} = \frac{Z'_{(\omega)}}{\omega\,|Z_{(\omega)}|^2} \qquad \text{(S6)}$$

S6. Performance evaluation in different operating temperatures

In temperature variation experiments, the 3-electrode evaluations of electrochemical performance were conducted with LiTFSI electrolytes in different concentrations, varying from "salt-in-water" (1 m) to "water-in-salt" (5 and 20 m). The evaluating temperatures are including -5, 0,10, 25, 40, and 60 °C, respectively. The ESW determination was conducted *via* CV and EIS was conducted for charge storage mechanisms interpretation. Three replicates for each electrolyte concentration were performed for preciseness of the results.

<u>Determination of ESW in temperature variation experiments</u>

Prior to the full-range determination of electrochemical stability window which includes both negative and positive potential. The individual negative and positive are evaluated and used as a negative and positive limit of the potential window. In the case of 1 m, the electrolyte freezes at a lower temperature than 0 °C. Therefore, data cannot be collected at – 5 °C. For the rest, their CVs are illustrated in Figure S8 below. The ESW for each case is summarized in Table S7 and also labeled in each graph. The diluted electrolytes provide the narrower ESW and also exhibit the reaction at lower potential. On the other hand, the WIS electrolytes show the wider potential window and a delay of reaction.

Table S7. Summary of ESW at each temperature for MoS_2-LiTFSI system.

**Temperature (°C)**	**1 m**	**5 m**	**20 m**
- 5	N/A	- 0.3 to 0.7 (1.0 V)	- 0.2 to 0.8 (1.0 V)

0	- 0.1 to 0.5 (0.6 V)	- 0.3 to 0.7 (1.0 V)	- 0.2 to 0.8 (1.0 V)
10	- 0.1 to 0.5 (0.6 V)	- 0.2 to 0.6 (0.8 V)	- 0.2 to 0.8 (1.0 V)
25	- 0.1 to 0.5 (0.6 V)	- 0.2 to 0.6 (0.8 V)	- 0.2 to 0.8 (1.0 V)
40	- 0.1 to 0.5 (0.6 V)	- 0.2 to 0.6 (0.8 V)	- 0.2 to 0.7 (0.9 V)
60	- 0.1 to 0.3 (0.6 V)	- 0.2 to 0.6 (0.8 V)	- 0.2 to 0.6 (0.8 V)

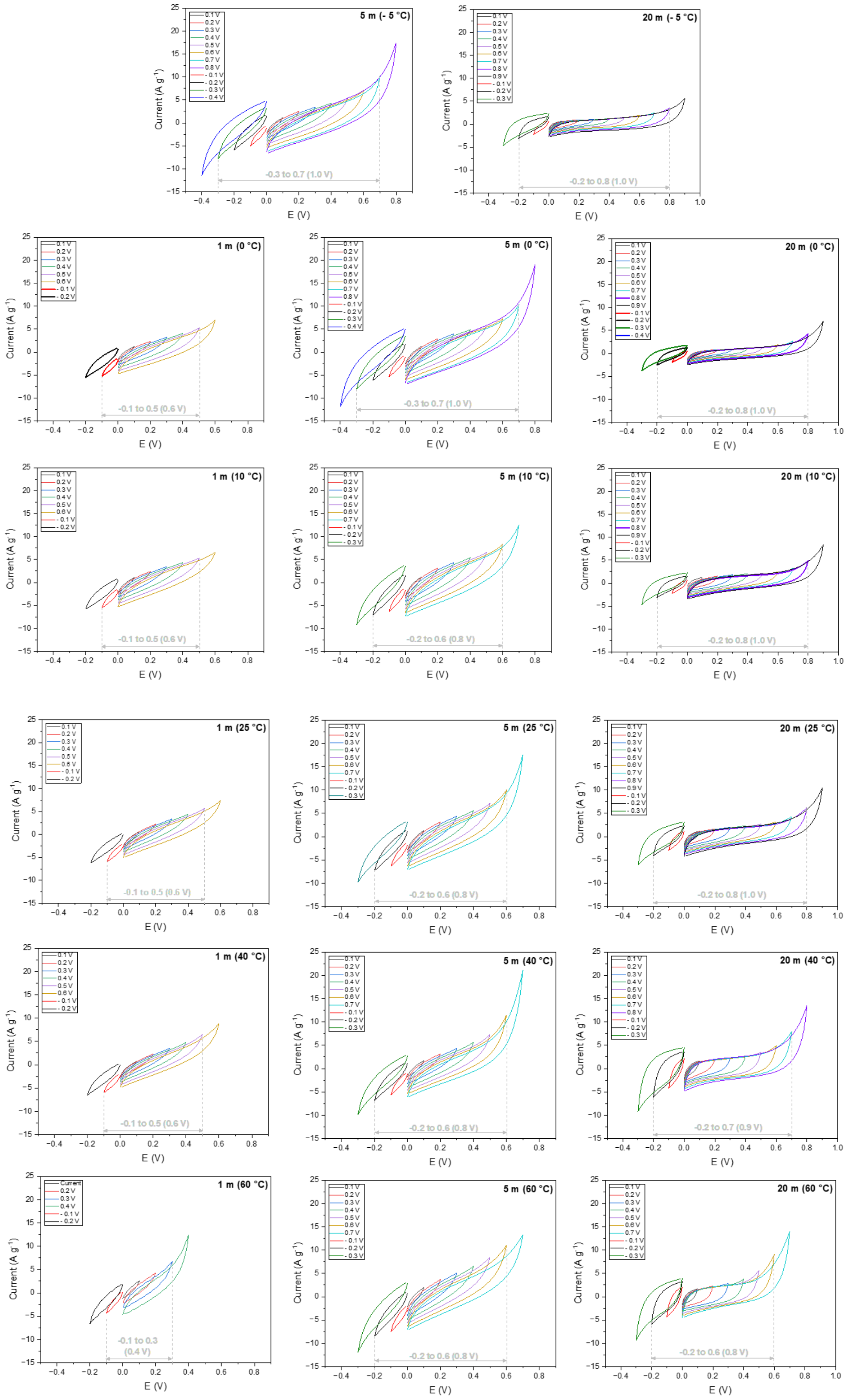

Figure S8. Electrochemical stability window determination in temperature variation experiment (-5, 0, 10, 25, 40, 60 °C). Note that the case of 1 m cannot be measure as the electrolyte freezes at a lower temperature than 0 °C.

Determination of specific capacitance in temperature variation experiment

In this section, the specific capacitance of the 17 conditions is calculated based on Equation S4 as mentioned in section S5. All the evaluation is done at scan rate of 100 mV s^{-1}, illustrated in Figure 3B in the main text. The calculated specific capacitance is listed in Table S8 below.

Table S8. Numerical data of area integration and *Cs* value calculation.

**Temperature**	***Cs* (F g^{-1})**		
**(°C)**	**1 m**	**5 m**	**20 m**
- 5	N/A	58.7 ± 2.3	27.7 ± 1.5
0	29.3 ± 2.0	59.8 ± 1.8	24.2 ± 1.3
10	25.0 ± 2.0	54.6 ± 2.3	29.5 ± 1.5
25	22.8 ± 2.0	50.6 ± 3.0	36.9 ± 1.8
40	23.6 ± 4.0	46.5 ± 3.5	48.7 ± 2.0
60	25.8 ± 5.6	54.1 ± 5.0	53.2 ± 2.0

EIS evaluation in temperature variation experiments

Figure S9 illustrated all the EIS measurements for 1, 5, and 20 m tested at different temperatures of -5 , 0, 10, 25, 40 and 60 °C. Since the trend of 1 and 5 m is quite similar, the data of 5 m are used as the representative and compared to 20 m in a main text. Considering Nyquist plot of 1 and 5 m, the overall impedance for both solutions is similar. For temperature dependency, the increased temperature decreases resistance in the system, especially the part of imaginary resistance. As in the inset, the semi-circle expands as the temperature increases, indicating an increase in charge-transfer resistance with increasing temperature. This phenomenon is in a small degree of expansion in the diluted solution of 1 m, but the change of overall impedance is found also in the diluted solution. The change of solution dynamic might be controlled by the amount of free water. Considering the Bode phase shift plot, the resistive component becomes more significant compared to capacitive component. The 5 m LiTFSI electrolyte shows relatively resistive interfacial behavior and slower kinetics, especially pronounced at lower temperatures (-5 and 0 °C).

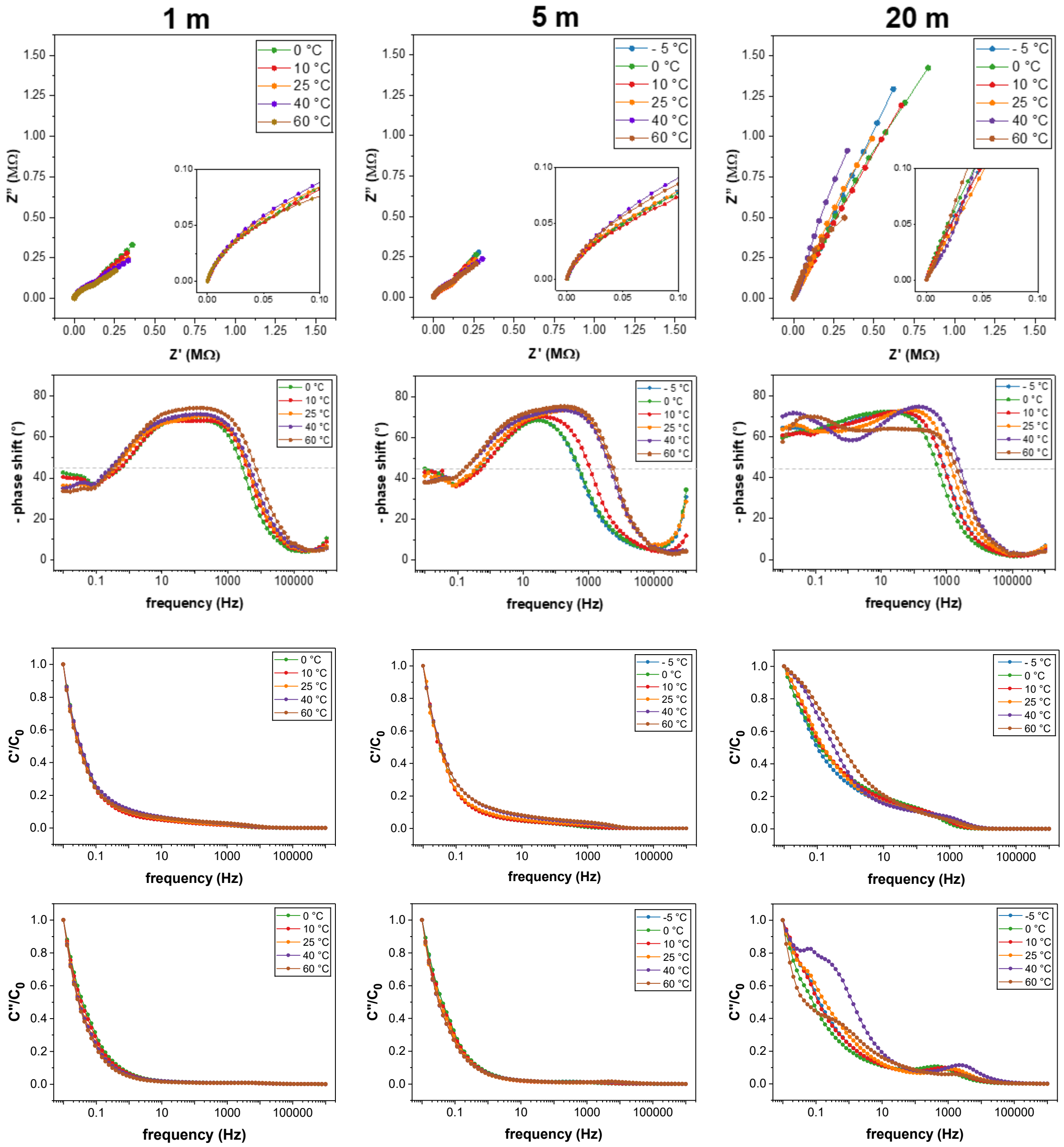

Figure S9. EIS evaluation on electrochemical performance of MoS_2 electrode at 5 m and 20 m LiTFSI electrolyte at different temperatures: (A) Nyquist plot of 5 m electrolyte, (B) phase shift of 5 m electrolyte, the results of 20 m electrolytes, including Nyquist plot (C), Bode phase shift plot (B), Real capacitance (C), and Imaginary capacitance (F), respectively.

S7. Performance evaluation in different variations of Li anions

For anion variation experiment, the electrochemical performance evaluation of Li-ion electrolytes varying the anion type was also performed in a three-electrode system of the same electrode material, MoS_2. The concentration of electrolytes was selected at 1 m of salt-in-water condition and the maximum possible concentration before recrystallization of each Li salt. Four types of Li salts were selected in this study, including Lithium bis(trifluoromethanesulfonyl)imide (LiTFSI), Lithium nitrate ($LiNO_3$), Lithium sulfate (Li_2SO_4), Lithium chloride (LiCl). The evaluation was conducted in cyclic voltammetry for ESW evaluation and varying scan rate for specific capacitance determination. EIS was also conducted to study the different frequency response of the system with their ion movement interpretation. Prior to the electrochemical evaluation, the electrolyte physiochemical properties, including electrical conductivity, viscosity, and pH, are measured and listed in Table S9 and S10.

Table S9. Physiochemical properties of various Li-ion type electrolytes (at 25 °C).

**Electrolytes**	**Electrical Conductivity ($mS\ cm^{-1}$)**		**Kinematic viscosity (cSt)**	
	**1 m**	**max conc***	**1 m**	**max conc***
**LiTFSI**	28.13 ± 0.06	10.05 ± 0.01	1.22 ± 0.00	29.11 ± 0.34
**$LiNO_3$**	57.39 ± 0.09	81.34 ± 0.07	1.02 ± 0.01	5.70 ± 0.05
**Li_2SO_4**	62.18 ± 0.48	57.57 ± 0.22	1.59 ± 0.02	4.09 ± 0.13
**LiCl**	61.01 ± 0.17	47.17 ± 0.29	1.09 ± 0.01	12.62 ± 0.07

Table S10. pH of various Li-ion type electrolytes (at 25 °C).

**Electrolytes**	**pH**	
	**1 m**	**max conc***
**LiTFSI**	7.22 ± 0.03	6.26 ± 0.01
**$LiNO_3$**	7.19 ± 0.04	5.07 ± 0.12
**Li_2SO_4**	7.06 ± 0.00	6.74 ± 0.07
**LiCl**	7.01 ± 0.01	3.93± 0.09

*max concentration in this study: LiTFSI 20 m, $LiNO_3$ 20 m, Li_2SO_4 3 m, and LiCl 20m

Determination of ESW in anion variation experiment

With the same consideration of determining the electrochemical stability window of 1, and max concentration before crystallization and possible in this experiment: which are 20 m for LiTFSI, $LiNO_3$, and LiCl, except for Li_2SO_4 which is prepared at 3 m. The ESW was determined with the same manner as the previous experiment. The results are illustrated in Figure S10.

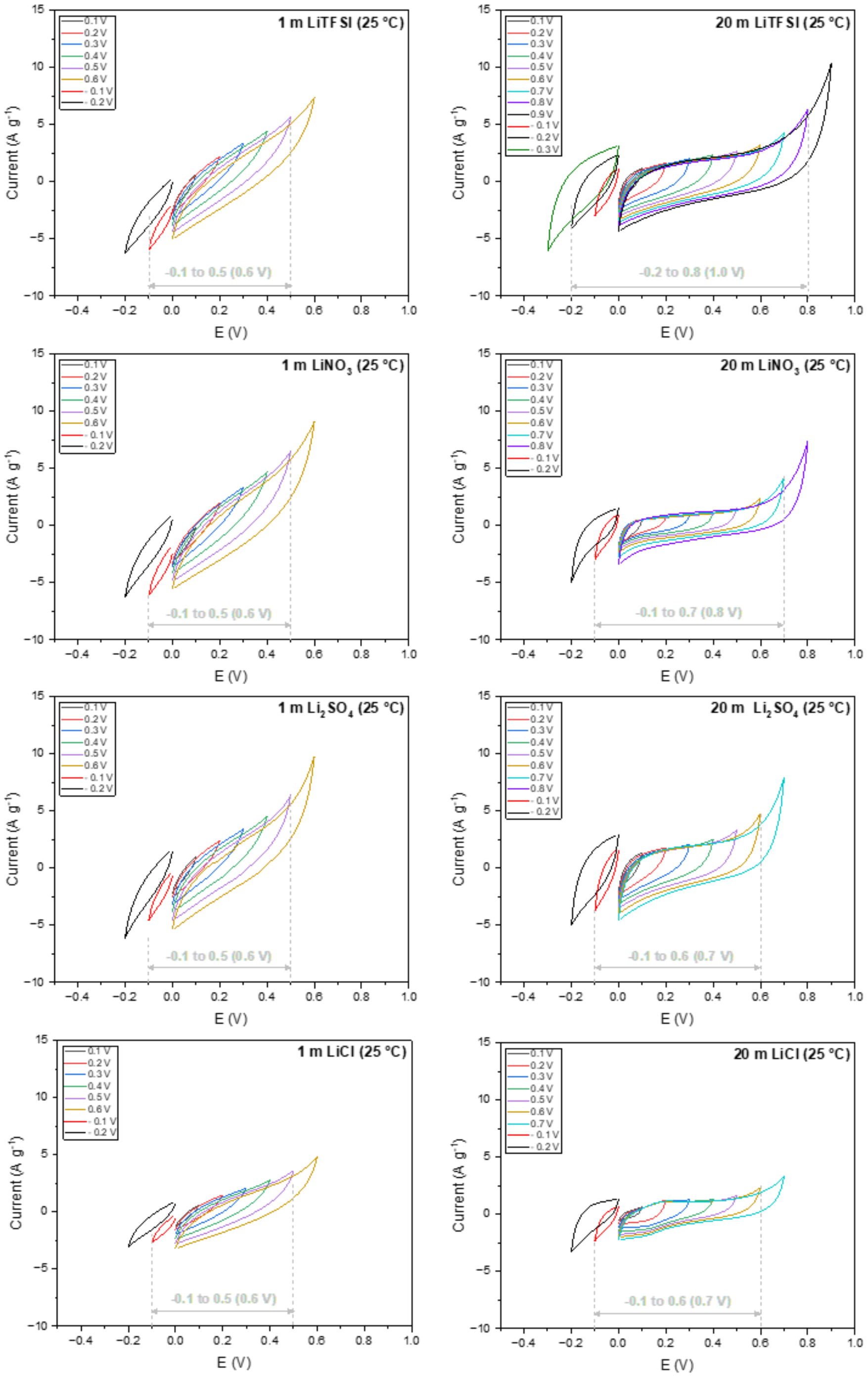

Figure S10. ESW determination in anion variation experiment.

Table S11. Summary of ESW at each temperature for MoS_2-LiTFSI system.

Temperature (°C)	1 m	Max concentration*
LiTFSI	- 0.1 to 0.6 (0.7 V)	- 0.2 to 0.8 (1.0 V)
$LiNO_3$	- 0.1 to 0.5 (0.6 V)	- 0.1 to 0.7 (0.8 V)
Li_2SO_4	- 0.1 to 0.5 (0.6 V)	- 0.1 to 0.6 (0.7 V)
LiCl	- 0.1 to 0.5 (0.6 V)	- 0.1 to 0.6 (0.7 V)

<u>Determination of specific capacitance in anion variation experiment</u>

In this section, the specific capacitance of four different electrolytes is calculated based on Equation S3 as mentioned in section S3. All the evaluation is done at scan rate of 100 mV s^{-1}. Their CVs are illustrated in Figure 5A in the main text. The calculated specific capacitance is listed in Table S12 below and shown in Figure 5C.

Table S12. Numerical data of area integration and Cs value calculation (anion variation experiment).

**Electrolyte**	C_S **(F g⁻¹)**	
	**1 m**	**max concentration***
LiTFSI	32.6	30.4
$LiNO_3$	18.1	30.7
Li_2SO_4	35.6	47.9
LiCl	15.6	24.9

*Max concentration in this study: LiTFSI 20 m, $LiNO_3$ 20 m, Li_2SO_4 3 m, and LiCl 20 m

EIS evaluation in anion variation experiments

Figure S11 shows the comparison of EIS measurement in anion variation experiments. The diluted system reveals the similarities between anion types. The slightly different are observed in Nyquist plot. LiTFSI demonstrates the best performance in charge transfer and the poorest charge transfer performance is LiCl. For Bode phase shift, the difference is clearly observed at mid-range frequency (1 – 1kHz). The time constants are implied from the frequency of the minimum phase angle peak in the mid-frequency region. The value from smallest value (faster kinetics) to largest time constant (slower kinetic): LiTFSI(fastest) → $LiNO_3$ → LiCl → Li_2SO_4 (slowest).

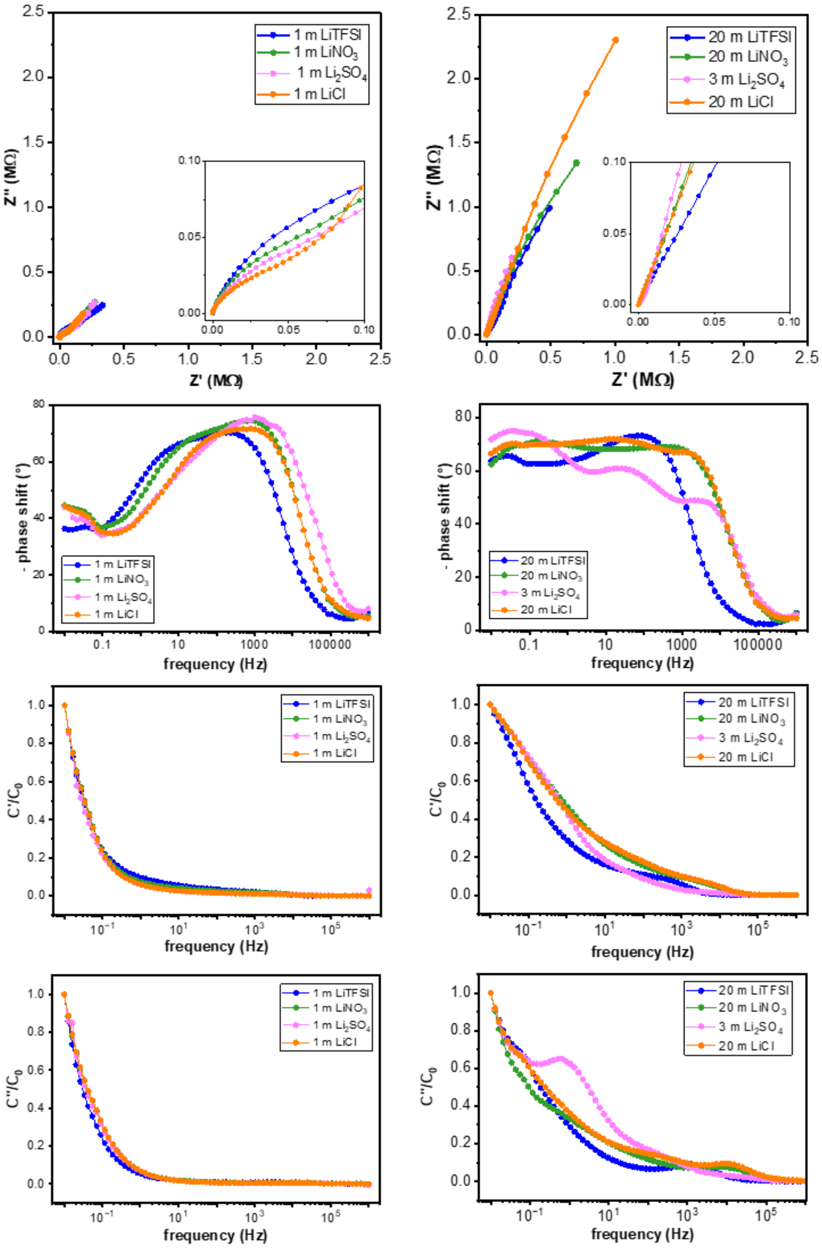

Figure S11. Full EIS data for the anion variation experiment.

References

(1) Voshell, A.; Terrones, M.; Rana, M. Thermal and Photo Sensing Capabilities of Mono- and Few-Layer Thick Transition Metal Dichalcogenides. *Micromachines* **2020**, *11* (7). https://doi.org/10.3390/mi11070693.

(2) Bunpheng, A.; Chavalekvirat, P.; Tangthana-umrung, K.; Deerattrakul, V.; Nueangnoraj, K.; Hirunpinyopas, W.; Iamprasertkun, P. A Comprehensive Study of Affordable "Water-in-Salt" Electrolytes and Their Properties. *Green Chem. Eng.* **2024**. https://doi.org/10.1016/j.gce.2024.06.004.
